# Spatiotemporal Communication in Artificial Cell Consortia for Dynamic Control of DNA Nanostructures

**DOI:** 10.1021/acscentsci.4c00702

**Published:** 2024-07-22

**Authors:** Antoni Llopis-Lorente, Jingxin Shao, Jordi Ventura, Bastiaan C. Buddingh′, Ramón Martínez-Máñez, Jan C. M. van Hest, Loai K. E. A. Abdelmohsen

**Affiliations:** † Department of Chemical Engineering and Chemistry, Institute for Complex Molecular Systems, Department of Biomedical Engineering, Eindhoven University of Technology, PO Box 513, 5600 MB Eindhoven, The Netherlands; ‡ Instituto Interuniversitario de Investigación de Reconocimiento Molecular y Desarrollo Tecnológico (IDM), Universitat Politècnica de València, Universitat de València, Camino de Vera s/n, 46022 València, Spain; § CIBER de Bioingeniería, Biomateriales y Nanomedicina (CIBER-BBN), Instituto de Salud Carlos III, 28029 Madrid, Spain

## Abstract

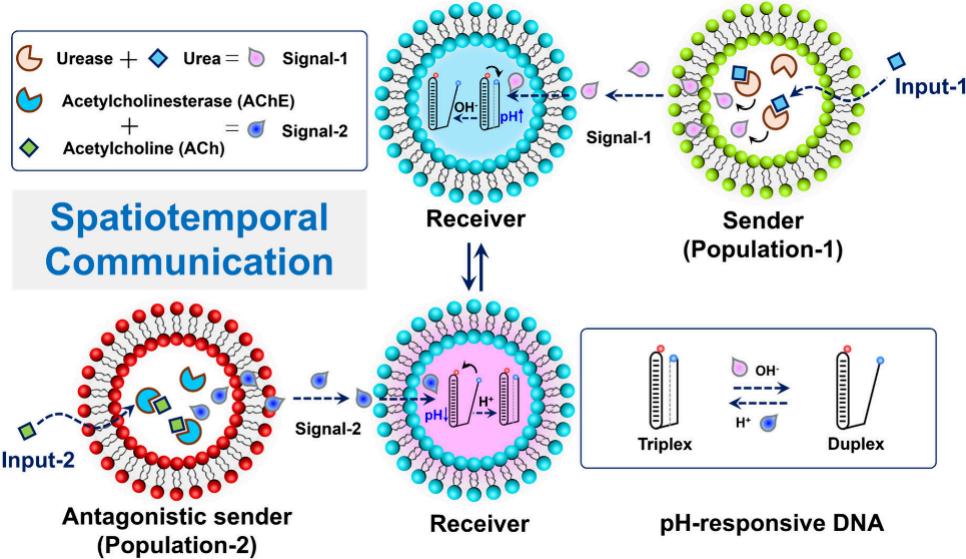

The spatiotemporal orchestration of cellular processes is a ubiquitous phenomenon in pluricellular organisms and bacterial communities, where sender cells secrete chemical signals that activate specific pathways in distant receivers. Despite its importance, the engineering and investigation of spatiotemporal communication in artificial cell consortia remains underexplored. In this study, we present spatiotemporal communication between cellular-scale entities acting as both senders and receivers. The transmitted signals are leveraged to elicit conformational alterations within compartmentalized DNA structures. Specifically, sender entities control and generate diffusive chemical signals, namely, variations in pH, through the conversion of biomolecular inputs. In the receiver population, compartmentalized DNA nanostructures exhibit changes in conformation, transitioning between triplex and duplex assemblies, in response to this pH variation. We demonstrate the temporal regulation of activated DNA nanostructures through the coordinated action of two antagonistic sender populations. Furthermore, we illustrate the transient distance-dependent activation of the receivers, facilitated by sender populations situated at defined spatial locations. Collectively, our findings provide novel avenues for the design of artificial cell consortia endowed with programmable spatiotemporal dynamics through chemical communication.

## Abstract 2

Consortia of sender and receiver artificial cell mimics are designed to orchestrate dynamic reconfiguration of DNA nanostructures upon transformation of chemical fuels, with spatiotemporal control.

## Introduction

Living cells employ chemical communication to transmit and process information from the environment in a modular manner. Sender cells secrete diffusive signals such as neurotransmitters, hormones, and morphogens to exert a certain effect in receiver cells.^1^ Upon sensing input signals, biological (nano)architectures in receiver cells undergo structural changes that articulate the cellular response. For instance, protein switches alter their shape upon ligand binding, chemical modification, or a change in the environment.^2,3^ Control of protein (e.g., caspase dimerization in response to inflammation triggers) and DNA (e.g., triplex/duplex transitions in response to genetic factors) structural organization is key to modulate biological processes.^4^ Specifically, triplex DNA structures are ubiquitously found in cellular genomes, especially at promoter regions, and impede the assembly of transcriptional machinery. Triplex-to-duplex transitions in response to internal and external stimuli (e.g., temperature) activate cellular processes such as mRNA synthesis.^5−8^ Importantly, chemical communication allows the dynamic control of biological processes in space and time.^9^

In artificial cell research, chemical communication holds potential as the basis to provide fundamental insights in the functioning of living cells, control over collective cell behavior, and future technological applications.^10−13^ Efforts to establish communication between artificial cells have recently been reviewed.^14^ In the past few years, several examples of artificial cell communication have been reported, based on the incorporation of processing machinery (enzymes, transcription–translation extracts, or DNA-strand displacement reactions) in different compartments (lipid vesicles, proteinosomes, coacervates, etc.).^15−23^ Most of these examples rely on (i) sender cells that produce or release a messenger and (ii) receiver cells that sense the messenger and produce an output molecule (a fluorescent product or release of an entrapped cargo). In a recent report, the exchange of substrates between enzyme-functionalized liposomes was leveraged to induce enhanced motility of receivers.^24^ Yet, achieving programmability over the communication process to control collective behavior is still an underexplored field of science,^25^ which holds enormous potential toward the development of smart materials and synthetic tissues. For example, external control over the communication processes has been demonstrated via the use of light-activable ligands.^26,27^ Control over protocell arrangements into microarrays has been achieved using acoustic trapping.^28^ Recently, oscillatory behavior has been demonstrated based on buoyant enzyme-powered protocells^29^ and based on communities of artificial and bacterial cells able to drive environmental pH changes.^30^ However, despite recent progress, the engineering of artificial cell consortia that exhibit spatiotemporal self-regulation of compartmentalized biological nanostructures remains unexplored.

**Scheme 1:**
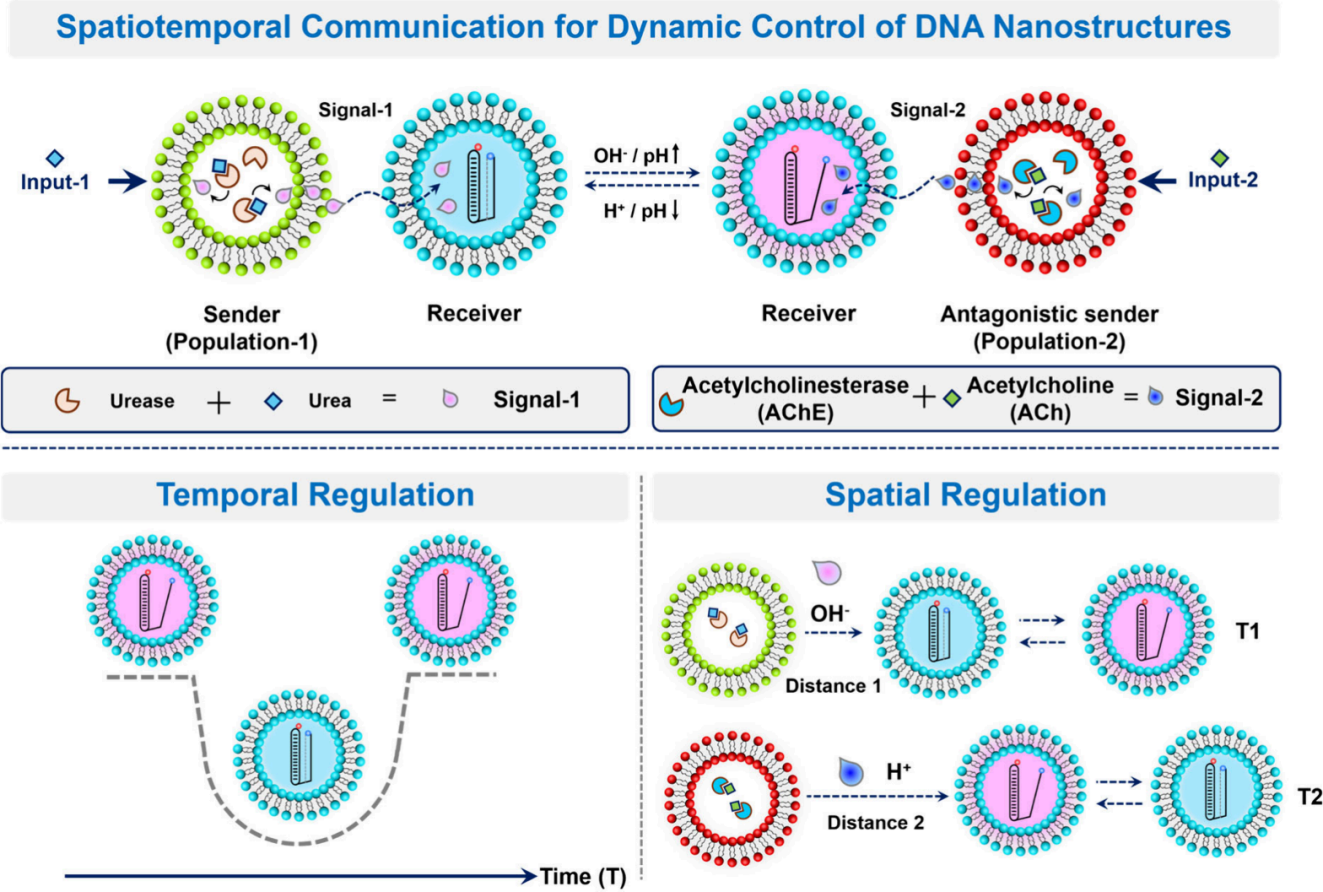
Design of Spatiotemporal Communication in Artificial Cell Consortia for Dynamic Control of DNA Nanostructures. (Top) Signaling from sender population-1 induces triplex-to-duplex transition of compartmentalized DNA nanostructures in the receivers, whereas signaling from the antagonistic sender population-2 induces the opposite (duplex-to-triplex) conformational change of DNA. (Bottom) Out-of-equilibrium temporal regulation of DNA nanostructures is orchestrated by signaling from the two sender populations, returning the DNA nanostructure to its initial conformation after a certain time. Spatial regulation is enabled based on the distance of the receivers to the sender populations.

In this paper, we demonstrate the engineering of chemical communication between artificial cells to achieve autonomous spatiotemporal control over compartmentalized DNA nanostructures (Scheme 1). In particular, sender population-1 is loaded with urease enzyme, and upon input of urea (trigger of the communication), ammonia is produced and diffuses as a molecular messenger to the receiver cells. The receiver population is equipped with pH-responsive DNA nanostructures (Figure S1) that switch from triplex to duplex conformation in response to the pH increase induced by ammonia (signal) which is produced by the senders. The antagonistic sender population-2 (loaded with acetylcholinesterase) is assembled to induce the opposite transition (duplex to triplex) of the DNA nanostructures in the presence of acetylcholine as a molecular input, which upon enzymatic hydrolysis leads to a pH decrease. By placing the sender and receiver populations at specific locations, temporal- and distance-dependent control over the compartmentalized DNA nanostructures is then demonstrated. This communication strategy is generic and, given the versatility and functionality of DNA nanostructures^31−37^ and enzymes, allows extension to design diverse programmable communication networks.

## Results and Discussion

Among the different artificial cell platforms available to construct our communication network, we opted for the use of giant unilamellar vesicles (GUVs) because of their semipermeable membrane and facility to encapsulate macromolecules.^11,38,39^ We prepared micrometer-sized (ca. 20 μm) GUVs based on the droplet transfer method from a mixture of phospholipids (DOPC, POPC) and cholesterol (35/35/30 molar ratio), to which an aqueous aliquot containing the molecular cargo (enzymes or DNA nanostructures) was added, followed by emulsification (see SI for details). In the first step, we studied the preparation of urease-loaded GUVs (Figures S2–S4). In order to confirm membrane permeability to urea (trigger of the communication), we evaluated the kinetics of urea transformation (lumen basification due to urease-mediated ammonia production) in urease-loaded GUVs (Figure S4). We compared GUVs with α-hemolysin pores (highly permeable membrane to allow the direct pass-through of chemicals) and GUVs without α-hemolysin (semipermeable membrane). In both cases, upon addition of urea to the external medium of urease-loaded GUVs, an increase in pH was observed in the GUV lumen, thus indicating the permeation of urea through the lipid membrane in line with previous studies.^40^ Moreover, the addition of α-hemolysin (enabling direct diffusion of urea to the lumen) was observed to result in faster kinetics, which indicates that the rate of urea transformation is partially slowed by the GUV membrane (in the absence of α-hemolysin). This effect may potentially be leveraged to modulate enzymatically driven processes based on tuning membrane permeability. In addition, we demonstrated that the rate of ammonia (signal) production could be modulated by adjusting the amount of enzyme incorporated in the GUV lumen during the preparation process (Figure S4c). Altogether, these results confirmed the ability of sender GUVs to recognize urea and produce ammonia.

To prepare receiver GUVs, we selected DNA nanostructures that are known to be responsive to pH. Such structures are based on a DNA nanoswitch, previously reported by Ricci’s group,^41^ that contains an intramolecular DNA hairpin, adopting a triplex structure stabilized by Watson–Crick and pH-dependent parallel Hoogsteen interactions.^42^ In particular, the DNA nanostructures we used comprised a 45-base single-stranded DNA, labeled with Cy3 on position 19 (loop of the hairpin duplex) and with Cy5 on the 3′-end (triplex-forming tail). Hoogsteen interactions between the triplex-forming tail and the duplex structure are disrupted at basic pH; thus the nanostructure adopts a triplex conformation at acidic pH and a duplex conformation at basic pH. The Cy3-Cy5 FRET pair allows to monitor the conformation of DNA by fluorescence upon excitation of Cy3, as Cy3 and Cy5 are in close proximity in the triplex conformation (resulting in a low Cy3–Cy5 fluorescence ratio), and they are separated in the duplex conformation (resulting in a high Cy3–Cy5 fluorescence ratio) (Figure S5). Upon encapsulation of the DNA in GUVs, Cy3 emission and Cy5 (FRET) emission were simultaneously collected at 550–620 and 640–715 nm, respectively, upon excitation of the donor fluorophore (i.e., Cy3) at 530 nm (Figures S6 and S7). In control experiments, we compared the response of DNA-nanoswitch-loaded receiver GUVs with the response of GUVs loaded with control DNA (Cy3- and Cy5-labeled random sequences) (Figures S6–S9). Whereas the output (Cy3–Cy5 ratio) remarkably increased upon addition of ammonia for receiver GUVs, control GUVs did not respond, thus confirming that the observed changes were governed by conformational changes of the DNA nanostructure.

**Figure 1:**
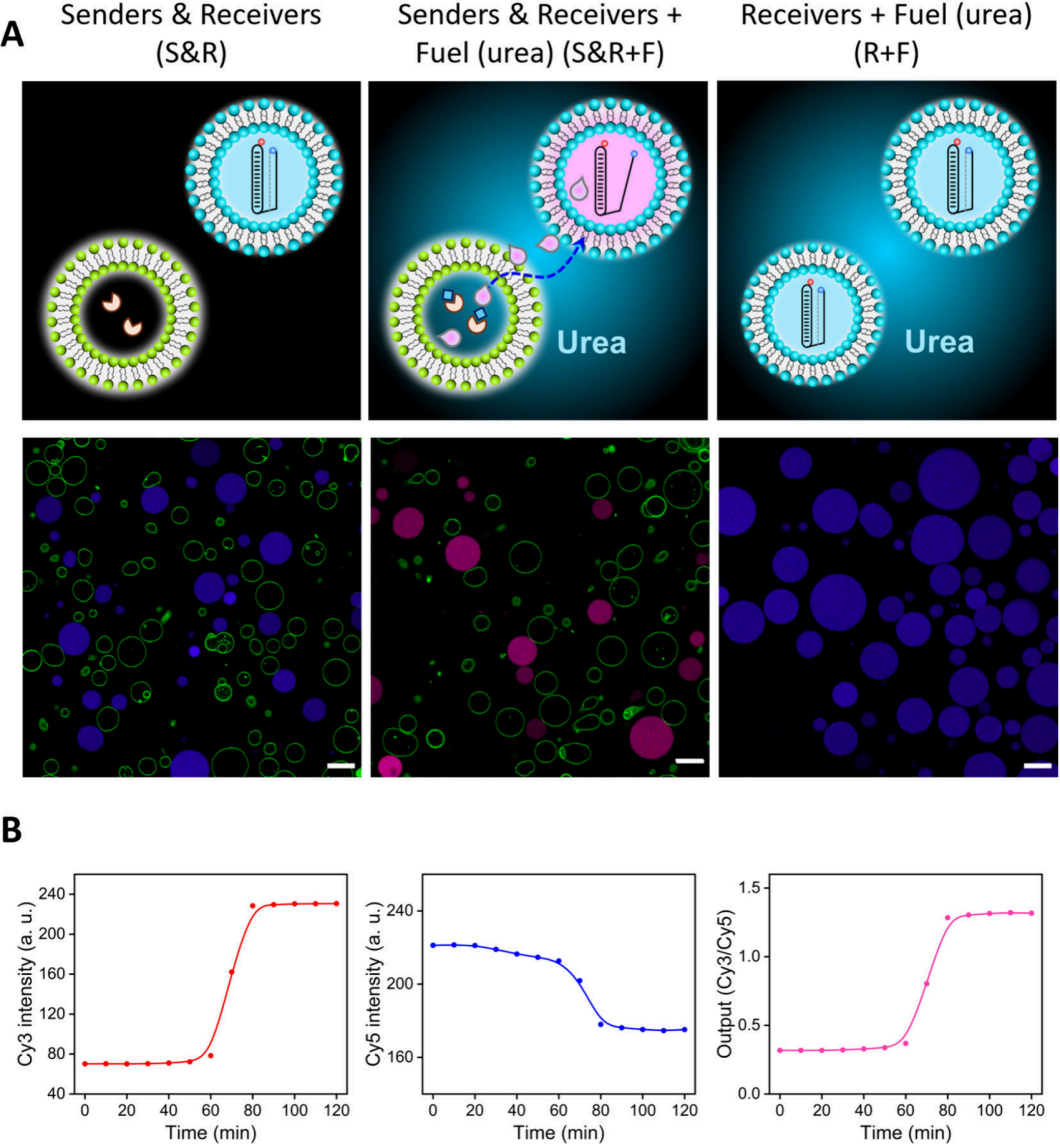
Chemical communication experiments between senders (urease-loaded, green membrane labeling) and receiver GUVs (DNA-loaded) in close proximity. **A)** Schematic and micrographs. Images show the merging of three channels: green (sender GUVs), red, and blue (Cy3 and Cy5 in receiver GUVs, respectively), which results in either blue (nonactivated) or pink (activated) receivers. (Left) In the absence of urea, the entrapped DNA nanostructure remains in the triplex state (blue); (middle) with input of urea (25 mM), ammonia-induced pH increase leads to the switching of the DNA nanostructure to the duplex conformation (pink); (right) in the absence of senders, there is no response. Scale bars represent 20 μm. **B)** Time-dependent fluorescence profiles of a selected (DNA-loaded) receiver GUV upon signaling from sender vesicles in the presence of urea. Changes in Cy3 and Cy5 emissions demonstrate a conformational change of the DNA nanostructure. Profiles of additional GUVs can be found in Figure S11.

We then set out to investigate the ability of sender and receiver GUVs to communicate by placing them together in a microscope chamber. The mixed populations were allowed to settle (10 min) followed by addition (or not) of urea as input of the communication network and co-incubation for 3 h. As shown in Figure 1 (and Figure S10), sender (green) and receiver (blue/pink) GUVs coexisted together in close proximity without clustering or coalescing. In the absence of urea, DNA nanostructures remained in the triplex state, as evidenced by the low Cy3 emission (red) and high Cy5 emission (blue), resulting in a blue color in the merged fluorescence channel. In contrast, when urea was added to the medium, receiver GUVs co-incubated with senders exhibited a high Cy3 emission (pink color in the channel merge), indicating DNA transition to the duplex conformation (Figure 1A). In addition, no response was observed when receiver GUVs were incubated in isolation upon addition of urea. This indicated that senders were able to process urea as input and emit ammonia (chemical messenger) to the external medium, which induced a change in receivers’ fluorescence via a pH-driven organization of the encapsulated DNA nanostructure. To gain insight into the dynamics of the process, we looked at the time-dependent fluorescence profiles of receiver GUVs upon signaling from sender GUVs in the presence of urea (Video S1). Inside a receiver GUV (Figures 1B and S11), a swift increase in Cy3 intensity and a concomitant decrease in Cy5 intensity were observed with time, demonstrating a conformational change of the DNA nanostructure. In further control experiments, when a nuclease enzyme was added to the external medium or coencapsulated in senders, we observed that the system remained functional; in contrast, when the nuclease was coencapsulated in receivers, communication was disrupted due to the hydrolysis of DNA by the nuclease (Figures S12–S14).

**Figure 2:**
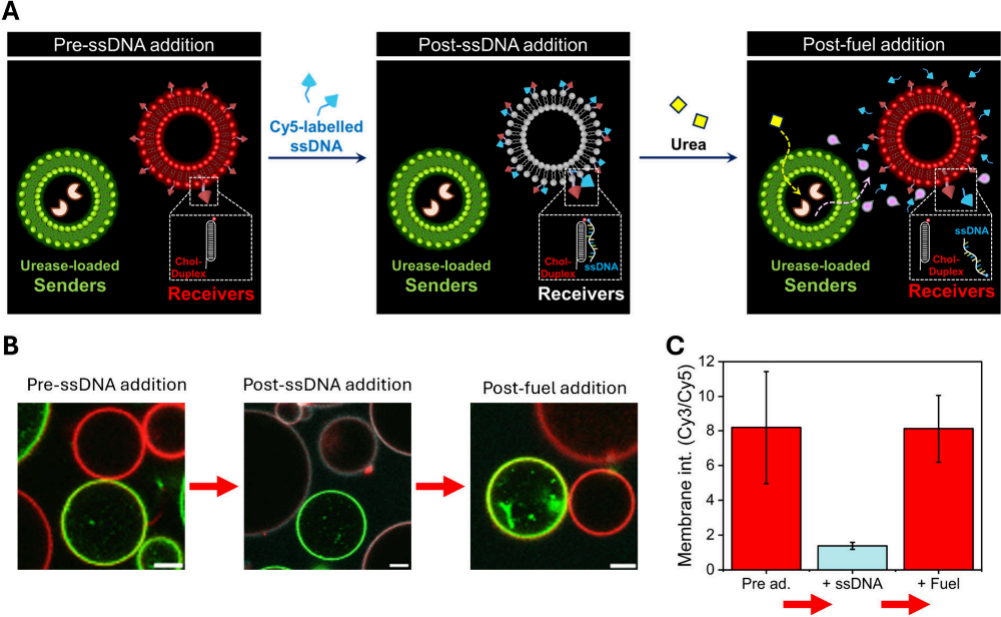
Capture and release of ssDNA from the membrane of duplex-functionalized receivers in an artificial cell consortium. **A)** Schematic representation of the experimental design and sequential processes and **B)** corresponding micrographs. Pre-ssDNA addition: urease-loaded senders (green) and receivers (red) functionalized with a cholesterol (chol)-duplex are co-incubated. Post-ssDNA addition: after addition and incubation with ssDNA (20 min), the formation of a triplex nanostructure on the membrane of duplex-functionalized receivers induces a decrease of the Cy3/Cy5 signal. Post-fuel addition: after subsequent incubation (70 min) upon addition of urea (25 mM), senders induce the release of ssDNA from receivers, leading to the recovery of Cy3 fluorescence. Scale bars represent 5 μm. **C)** Output (Cy3/Cy5 membrane intensity ratio) quantification for the different conditions. Data are plotted as mean ± s.d. (*N* ≥ 12 GUVs).

Additionally, we set out to demonstrate that DNA nanostructures could be assembled on the membrane of receiver GUVs and that the release of short DNA strands could be triggered by signaling from sender GUVs. To achieve this, we designed a Cy3-labeled 30-base DNA duplex tagged with a cholesterol moiety on its 5′-end. This cholesterol moiety enabled anchoring of the DNA duplex on the membrane of receivers (Figure 2A), during the preparation process. In addition, we employed a 10-base Cy5-labeled single-stranded (ss)DNA able to assemble on the duplex by Hoogsteen interactions, displaying a pH-dependent behavior (Figure S15). Preparation of receivers with a Cy3-labeled cholesterol (chol)-duplex resulted in the labeling of the lipid membrane, which was confirmed by confocal microscopy. Next, we mixed two populations (green and red membrane, respectively in Figure 2): (1) senders based on bodipy-labeled urease-loaded GUVs and (2) receivers based on duplex-functionalized GUVs. The consortium was imaged before (pre-ssDNA addition) and after (post-ssDNA addition) supplementation of the external medium with Cy5-labeled ssDNA. As shown in the post-ssDNA addition schematic and middle micrograph, a low Cy3/Cy5 intensity ratio on the receiver’s membrane was registered (resulting in a white membrane on the channel overlay). This indicated the capture of ssDNA on the membrane of duplex-functionalized GUVs by the formation of a triplex structure. As shown in the post-fuel addition schematic and right micrograph, upon subsequent addition of urea, (base) signaling from urease-loaded GUVs was able to induce the release of the single-stranded DNA from the receivers’ membrane, leading to a recovery of the Cy3 intensity on the receivers’ membrane. Thus, this set of experiments showcased the capture and release of short DNA strands in sender–receiver consortia.

**Figure 3:**
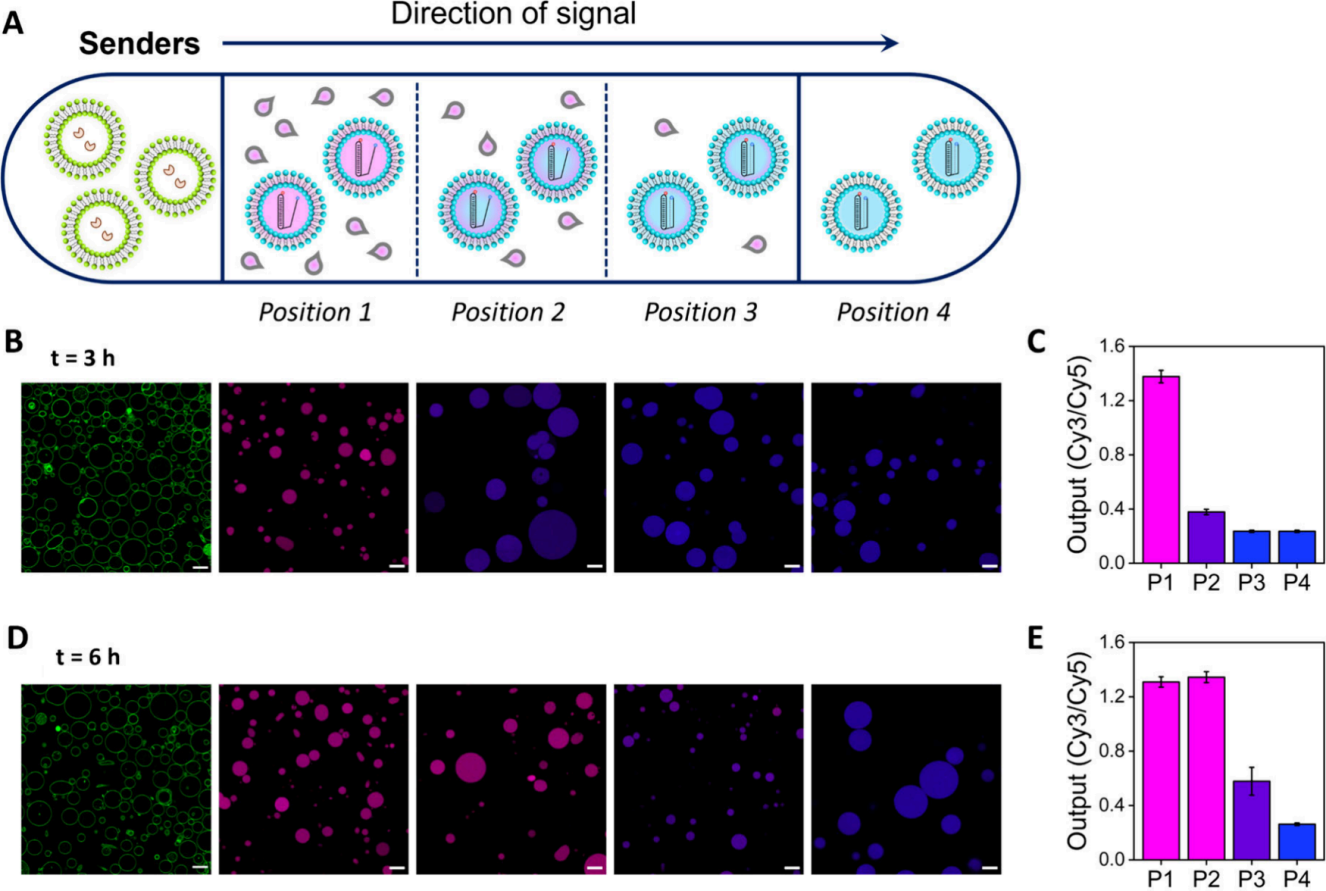
Sender GUVs communicate with distant receivers at different positions in a channel device. **A)** Schematic of the channel device and different positions measured by confocal microscopy. **B)** Micrographs with increasing distance of receivers to the sender population at *t* = 3 h and **C)** corresponding quantification of receiver output. **D)** Micrographs with increasing distance of receivers to the sender population at *t* = 6 h and **E)** corresponding quantification of receiver output. Activation occurs in a distance-dependent manner. Scale bars are 20 μm. Data are plotted as mean ± s.d. (*N* ≥ 8 GUVs).

Next, to demonstrate the possibility to activate receivers at different distances from senders (based on the diffusion of a secreted chemical signal), we employed a microfluidic channel device, as depicted in Figures 3 and S16 (dimensions 17 × 3.8 × 0.54 mm). The channel was loaded with DNA-nanoswitch-loaded receiver GUVs, and the reservoirs at the sides of the channel were filled with aqueous phase. Then, an aliquot of sender GUVs was gently deposited on one end, followed by addition of 1 μL of urea at the senders’ position (source point). Activation of receivers was measured as a function of their distance to the source point: at the middle (position 2, ∼8.5 mm) and opposite end of the channel (position 4, ∼16.5 mm) and at intermediate positions 1 (∼4.25 mm) and 3 (∼12.75 mm). After 3 h of incubation, senders were able to activate distal receivers at position 1, but activation was significantly weaker at position 2 and negligible at larger distances. With longer time (6 h), receivers’ activation was triggered at position 1 and also at position 2, whereas activation attenuated at position 3 and no activation was observed at position 4. Overall, these results showed that receivers’ activation can be triggered in a spatiotemporal manner depending on their distance to the senders.

To reversibly drive the opposite conformational change (duplex-to-triplex) in the DNA nanostructure, we prepared an antagonistic population of acetylcholinesterase-loaded GUVs. Acetylcholinesterase hydrolyzes the neurotransmitter acetylcholine into choline and acetic acid, thereby leading to acidification.^43^ Acetylcholine has been reported to need membrane carriers to permeate through lipid membranes.^44^ To ensure accessibility of the substrate to the enzyme, α-hemolysin was added to insert into the GUV membrane (Figure S17). To confirm enzymatic signaling, acetylcholinesterase-loaded senders and DNA-nanoswitch-loaded receivers were placed at pH 9 in a microscope chamber followed by the addition (or not) of acetylcholine. In the absence of acetylcholine (Figure S18), a high Cy3–Cy5 signal ratio was registered corresponding to the DNA nanostructure in the opened (duplex) state. In contrast, receivers showed a clear reduction in the Cy3–Cy5 ratio when co-incubated with senders upon addition of acetylcholine (Video S2 and Figure S18). As a control, no response was observed from receivers in the presence of acetylcholine but in the absence of senders. Thus, these results confirmed the ability of acetylcholinesterase-loaded GUVs to process acetylcholine from the environment and drive a pH-induced duplex-to-triplex transition in the receivers.

**Figure 4:**
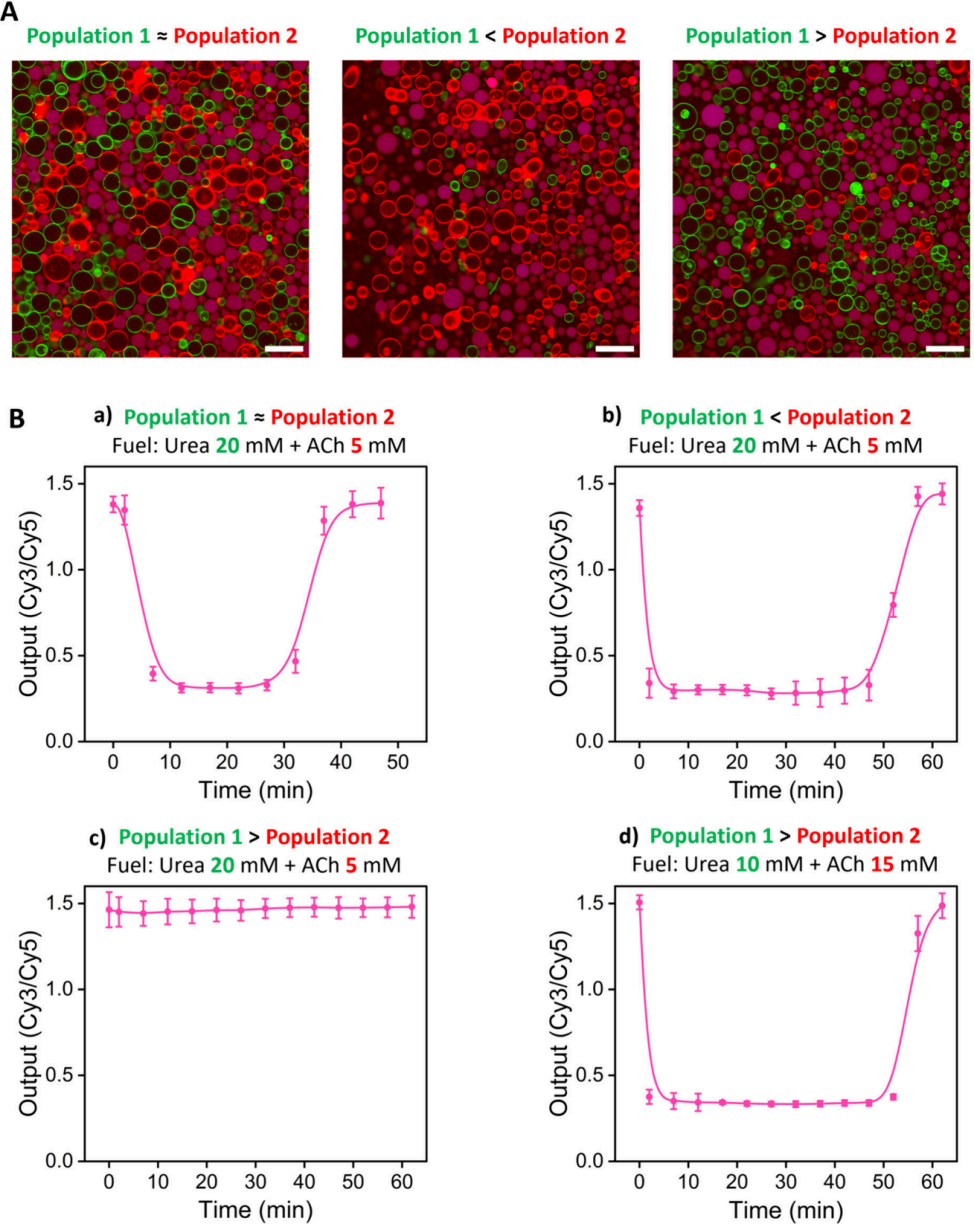
Communication in artificial cell consortia with two antagonistic sender populations drives dynamic out-of-equilibrium reorganization of DNA nanostructures in receivers. **A)** Micrographs of the different consortia composed of (left) equal proportion of sender population-1 (green membrane, urease-loaded) and sender population-2 (red membrane, acetylcholinesterase-loaded), (middle) major proportion of population-2 (red) vs population-1 (green), (right) major proportion of population-1 (green) vs population-2 (red). DNA-loaded receivers are shown in pink (overlay of Cy3 and Cy5 channels), corresponding to DNA nanostructure in the duplex conformation before substrate addition (*t* = 0). Representative time lapse micrographs are shown in Figure S20 and Video S3. Scale bars are 50 μm. **B)** Time-dependent profiles of receivers’ output under different conditions (a–d) upon simultaneous addition of urea and acetylcholine (ACh). Samples were initially set at pH 9, where the DNA nanostructure is in the duplex state. Temporal reduction of the Cy3/Cy5 ratio indicates a temporal transition to the triplex state. Data are plotted as mean ± s.d. (*N* = 14 GUVs).

In the next step, we aimed at evaluating the response of receivers upon the action of the two antagonistic sender populations (i.e., urease-loaded GUVs as sender population-1 and acetylcholinesterase-loaded GUVs as sender population-2). Sender population-2 was expected to act faster due to the high activity of native acetylcholinesterase (∼1000 U/mg) compared to native urease (∼50 U/mg). Thus, consortia composed of DNA-nanoswitch-loaded receivers and the two sender populations were set at basic pH at which the DNA nanostructure is in the duplex state. Three types of consortia were assembled containing (case a) the same proportion of GUVs from population-1 and population-2, (case b) a major proportion of population-2 vs population-1, and (case c) a major proportion of population-1 vs population-2 (Figure 4A). Then, urea and acetylcholine were simultaneously added (20 and 5 mM, respectively), and consortia were monitored over time (Figures 4B, S19, and S20). Urea was added in excess over acetylcholine to ensure that the system would return to basic pH. Under these conditions, receivers showed a swift transition to the triplex state in cases a and b, which was faster in case b due to having a major proportion of acetylcholinesterase-senders. Gratifyingly, receivers returned to their initial (duplex) state in both case a (Video S3) and b. The lifetime of the duplex state was longer in case b, which is attributed to having fewer urease-senders to drive the production of ammonia. In contrast to cases a and b, there was no significant change upon addition of substrates in case c (20 mM urea, 5 mM acetylcholine), which is ascribed to the faster action of the urease-based senders (in excess) over acetylcholinesterase-senders in this population. Yet, out-of-equilibrium behavior (duplex–triplex–duplex) in urease-overpopulated consortia was restored when the acetylcholine concentration was tripled (15 mM) and the urea concentration was reduced (10 mM). Altogether, this set of experiments highlights the possibility of designing artificial cell consortia able to exhibit autonomous transient behavior driven by two antagonistic populations in the presence of certain substrate concentrations.

**Figure 5:**
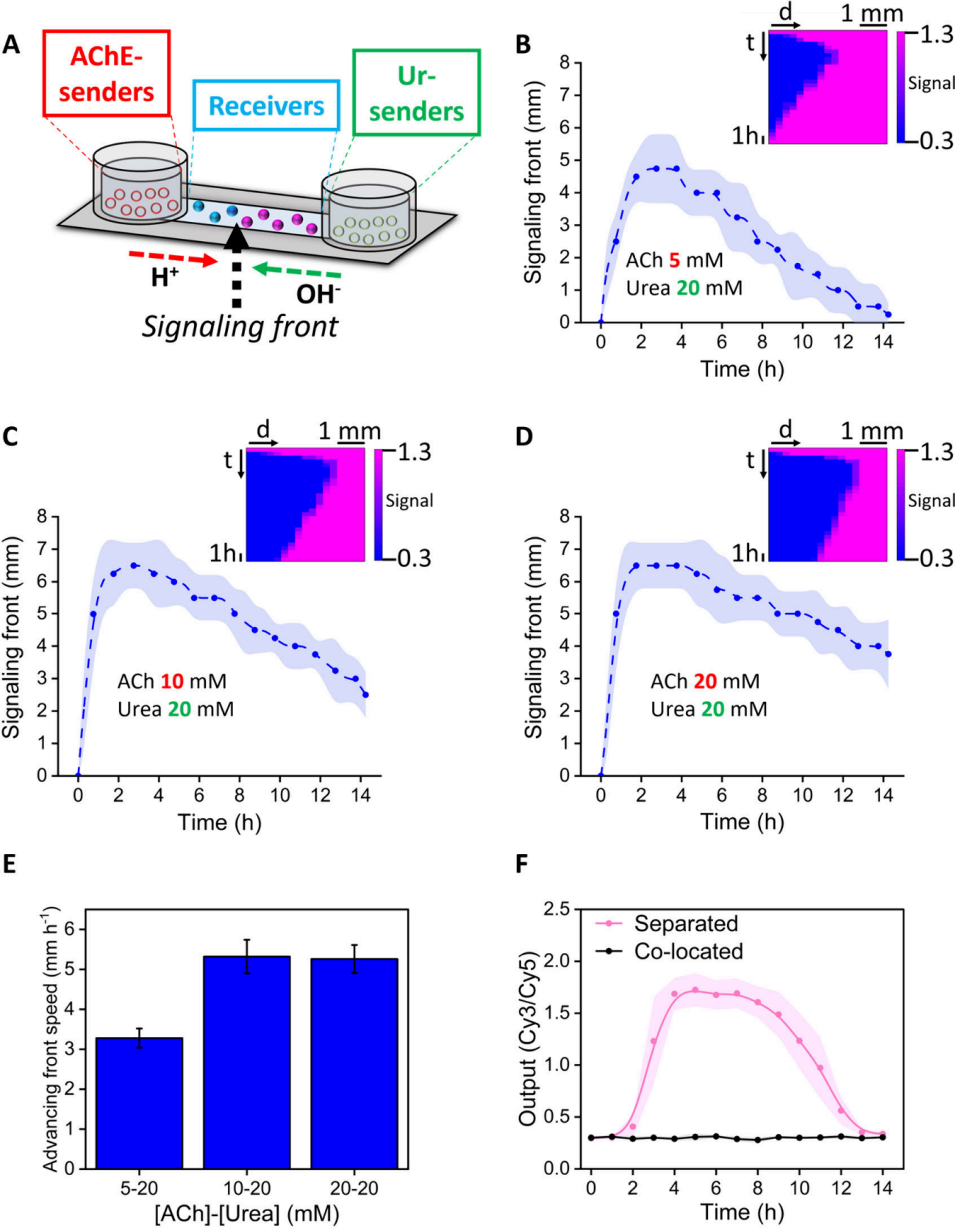
Spatiotemporal communication drives the transient activation of receivers. **A)** Schematic representation of the setup, where receivers are placed along a microfluidic channel. Acetylcholinesterase (AChE)-loaded senders and urease (Ur)-loaded senders are located at opposite ends. The system is initially set at pH 9, at which the DNA nanostructure is in the duplex state (pink). Signaling from the acid-producing AChE-senders induces the transition of receivers to the triplex state (blue). The position of the signaling front (furthest receiver in the triplex state (blue)) evolves with time, advancing in the channel during the first hours and receding as base signaling from Ur senders increases. **B–D)** Signaling front (furthest located receiver with DNA in the triplex state) as a function of their distance to the acid-producing AChE-senders. Insets depict heatmaps plotting receivers’ output along the length of the channel as a function of time. Acetylcholine (ACh) and urea were simultaneously added at the indicated concentration. **E)** Speed of the advancing front (receivers switched to the triplex state) under different substrate concentrations, as extracted by linear fitting of the distance vs time plots. **F)** Kinetics of Cy3/Cy5 ratio (initial pH = 5) indicate triplex–duplex–triplex transition for receivers when the two antagonistic sender populations were separated in the channel device. Imaged receivers were located near the urease-GUVs reservoir (1.5–3 mm distance). No response is observed when the two sender populations were colocated. As substrates, 5 mM urea and 20 mM acetylcholine were simultaneously added.

In a further step, we decided to evaluate the possibility of designing transient spatiotemporal activation patterns. For this, we placed acetylcholinesterase- and urease-senders in opposite reservoirs at the end of a microfluidic channel loaded with DNA-nanoswitch-loaded receivers in their duplex state (at pH 9) (Figure 5A). Then, acetylcholine and urea were added as inputs to start the signaling process. Time-lapse confocal imaging of subsequent positions along the channel was carried out to monitor the position of the signaling front (position of the furthest receiver activated by the acid-producing acetylcholinesterase-senders). Micrographs showed the transition to the triplex state of receivers as a function of their distance to the acetylcholinesterase population (Figures 5B–D, S21, and S22). At 5 mM acetylcholine and 20 mM urea, the (acidic) signal produced by acetylcholinesterase-senders advanced in the channel during the first couple of hours, enabling the activation of receivers located up to a 4–5 mm distance (Figure 5B). Then, after a lag time, (base) signaling from the urease-senders induced a progressive recession of the activation front and the return of the system to its initial state after 14 h. Interestingly, when we used a higher acetylcholine concentration (10 mM), the activation front advanced further in the channel (up to 6–7 mm) and the counteracting action of urease-senders could just induce the partial recession of the activation front (up to 2.5 mm) after 14 h (Figure 5C). Remarkably, the advancing front did not go further in the channel for the highest acetylcholine concentration (20 mM), but the receding front induced by urease-senders was slowed down due to the counteracting action of acetylcholinesterase-senders that have more substrate available. From these plots, we could extract the speed of the advancing and receding fronts at different substrate concentrations (Figures 5E and S23). As a control experiment, we confirmed that in the absence of the urease-senders the receivers did not return to their original state, and only the advance (but no recession) of the signaling front was observed (Figure S24). Furthermore, we were also able to induce the opposite behavior (triplex–duplex–triplex transition) when the system was first set at acidic pH in the receivers in proximity to the urease-senders, thanks to the delay of the acidic signal produced by distal acetylcholinesterase-senders, a behavior that could not be induced without spatial separation (Figures 5F, S25). Altogether, these results highlight the possibility to create spatiotemporal activation patterns using antagonistic sender populations. Transient spatiotemporal activation patterns are characteristic of cellular signaling in multicellular organisms, where molecules such as hormones and morphogens display dynamic concentration gradients that drive various biological processes in receivers.^9^ In this regard, the creation of spatiotemporal patterns using artificial cells is an important step toward mimicking the complexity of natural cellular signaling.

## Conclusions

In summary, we have demonstrated spatiotemporal communication in artificial cell consortia, enabling the dynamic control of DNA nanostructures. In particular, we prepared two antagonistic sender populations based on the encapsulation of antagonistic enzymes (urease and acetylcholinesterase) in GUVs, which could reversibly induce a pH-dependent triplex-to-duplex transition of a DNA nanostructure contained in receiver GUVs. Consortia composed of three different populations displayed temporal behavior that was dependent on the activity ratio between the two senders. In addition, we showed the creation of transient spatiotemporal activation patterns when receivers were placed in a microfluidic channel with antagonistic sender populations located at opposite ends. The design of spatiotemporal patterns based on chemical communication between artificial cells holds potential in different areas, such as the development of advanced drug delivery systems and smart materials. For instance, senders could potentially sense the levels of certain biomarkers in one area and emit diffusive chemical signals to dynamically activate drug delivery by distal receivers located in the target tissues. Different actuating mechanisms could be implemented in receivers; in particular, changes in DNA conformation could trigger protein synthesis or induce membrane deformations.^37^ In addition, dynamic communication mechanisms could be integrated into artificial tissues to develop dynamic smart materials that exhibit spatiotemporal changes in response to environmental conditions. Overall, our results open new avenues for engineering and investigating dynamic chemical communication processes using artificial cell mimics.

## Data Availability

Experimental details and additional data are available in the SI. Any other data supporting this article are available from the corresponding authors upon request.

## Supporting Information Available

Materials and methods, Figures S1 to S25, supplementary references (PDF)
Video S1: Urease-senders and DNA-receivers (AVI)
Video S2: Acetylcholinesterase-senders and DNA-receivers (AVI)
Video S3: Three population consortium with equal proportion of senders (AVI)
Transparent Peer Review report available (PDF)

## Supplementary Material

oc4c00702_si_001.pdf

oc4c00702_si_002.avi

oc4c00702_si_003.avi

oc4c00702_si_004.avi
