# Supplementary material for: Spatiotemporal Communication in Artificial Cell Consortia for Dynamic Control of DNA Nanostructures
Source: ACS Cent Sci. 2024 Jul 22;10(8):1619–28. doi: 10.1021/acscentsci.4c00702 (PMC11363350; doi:10.1021/acscentsci.4c00702)
Supplement: Supplementary file 1 — oc4c00702_si_001.pdf [file oc4c00702_si_001.pdf]

Supporting Information for

Spatiotemporal Communication in Artificial Cell Consortia for Dynamic Control of DNA Nanostructures

Antoni Llopis-Lorente,[abc] Jingxin Shao,[a] Jordi Ventura,[b] Bastiaan C. Buddingh′,[a] Ramón Martínez-Máñez,[bc] Jan C. M. van Hest,*[a] and Loai K. E. A. Abdelmohsen*[a]*

[a] Department of Chemical Engineering and Chemistry, Institute for Complex Molecular Systems, Department of Biomedical Engineering, Eindhoven University of Technology, PO Box 513, 5600 MB Eindhoven, The Netherlands. E-mail: j.c.m.v.hest@tue.nl, l.k.e.a.abdelmohsen@tue.nl.

[b] Instituto Interuniversitario de Investigación de Reconocimiento Molecular y Desarrollo Tecnológico (IDM), Universitat Politècnica de València, Universitat de València, Camino de Vera s/n, 46022 València, Spain. E-mail: anllolo2@upv.es.

[c] CIBER de Bioingeniería, Biomateriales y Nanomedicina (CIBER-BBN), Instituto de Salud Carlos III, Spain.

Materials and methods

Chemicals

1,2-dioleoyl-*sn*-glycero-3-phosphocholine (DOPC), 1-palmitoyl-2-oleoyl-glycero-3-phosphocholine (POPC), 1,2-distearoyl-*sn*-glycero-3-phosphoethanolamine-*N*-[biotinyl(polyethylene glycol)-2000] (DSPE-PEG), 23-(dipyrrometheneboron difluoride)-24-norcholesterol (Bdp-Chol), and 1,2-dioleoyl-*sn*-glycero-3-phosphoethanolamine-N-(lissamine rhodamine B sulfonyl) (DOPE-LRB) were provided by Avanti Polar Lipids. Paraffin oil (0.86 g/cm^3 at 20 °C) was from JT Baker. Cholesterol, glucose, sucrose, sodium chloride, urea, urease from *Canavalia Ensiformis* (Type III), acetylcholinesterase from *Electrophorus electricus* (Type VI-S), acetylcholine chloride, bovine serum albumin (BSA), alpha-hemolysin from *Staphylococcus aureus*, phosphate buffer saline pills, and deoxyribonuclease I from bovine pancreas (Type IV, 2000 KU/mg) were purchased from Sigma-Aldrich. Dextran-fluorescein-tetramethylrhodamine (dx-FITC/TMR) conjugate (70,000 g/mol, anionic) was obtained from Thermo Fisher Scientific.

All other reagents and solvents were of high-quality grade, purchased from commercial suppliers, and used without further purification. Ultrapure water ("Milli-Q") was obtained from a Merck Millipore Q-Pod system (≥ 18.2 MΩ) with a 0.22 μm Millipore Express 40 filter. Chambered microscope slides (18 wells, glass bottom) and channel slides (μ-slide VI 0.5 glass bottom) were purchased from Ibidi.

DNA sequences

DNA was synthesized and purified (HPLC) by Integrated DNA Technologies (Germany). Stock solutions were prepared at a concentration of 100 μM in ultrapure water and stored at -20 °C until use. Specific DNA sequences are listed below, where the bases in bold represent the loop for the duplex and triplex portions. Italics represent additional bases added to the switch region.

- *pH-responsive DNA nanoswitch* (adapted from Sánchez *et al.*)[1]:

5'-*TTTTTT* GAAGAAGGAA **TTT(Cy3)A** TTCCTTCTTC **GTTTG** CTTCTTCCTT(Cy5)-3'

- *Cholesterol-tagged duplex DNA*:

5'-(Chol) TTTTTT GAAGAAGGAA **TTT(Cy3)A** TTCCTTCTTC-3'

- *Single-stranded DNA (ssDNA):*

5'-CTTCTTCCTT(Cy5)-3'

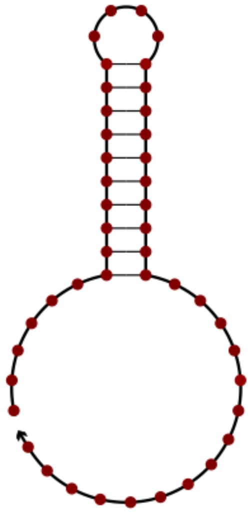

Figure S1. Structural description of the pH-responsive DNA nanoswitch sequence, using NUPACK.[2]

Instrumentation

Confocal laser scanning microscopy was performed using a Leica TCS SP5X for characterization and communication experiments. For spatiotemporal experiments with microfluidic channels, a Leica TCS SP8 was employed. For membrane experiments reported in Figure 2, and urea conversion kinetics (Figure S4), a Leica TCS DMi8 was employed. pH measurements were conducted using a FiveEasy Plus FEP20 pH meter (Mettler Toledo). Bulk fluorescence spectroscopy measurements were carried out using a JASCO FP-8500 spectrophotometer. Micrograph images were quantified and processed with Fiji, a program developed by the NIH and available as public domain software at https://imagej.net/Fiji.

Assembly of giant unilamellar vesicles (GUVs)

Our protocol for the preparation of GUVs is based the droplet transfer method.[3] Lipid stock solutions were prepared in chloroform and stored at -20 °C until use. Lipid solution aliquots were taken and mixed with 200 µL of paraffin oil to obtain a lipid mixture containing DOPC, POPC and cholesterol in a molar ratio of 35/35/30. For preparing pegylated liposomes, 1% of DSPE-PEG was added as well. When membrane labelling was needed, 0.06% of Bdp-Chol or DOPE-LRB were incorporated. Firstly, the lipid mixture in paraffin was heated at 80 °C for 30 min and cooled on ice for at least 15 min.

Next, 20 μL of inner phase solution (*vide infra*) was emulsified in 200 μL of lipid solution by strong vortexing for 25 s while turning the reaction tube to prevent sedimentation of the water droplet. Directly after, the emulsions were incubated on ice for 10 min. Subsequently, they were layered on top of 150 μL of pre-cooled outer phase (10 mM acetate buffer, 150 mM NaCl, 200 mM glucose) solution in a 1.5 mL plastic tube and immediately centrifuged at 4 °C for 20 min at 3300 rcf. The pH of the outer phase was adjusted to 5 or 9 as required for the experiment by addition of small amount of diluted HCl or NaOH. For preparing membrane duplex-functionalized GUVs, 2 μL of cholesterol-tagged duplex DNA (stock of 100 μM) were added to the outer phase solution. GUVs were harvested by puncturing the tube at the position of the GUV pellet and dripping the aqueous phase. To remove any non-encapsulated material, GUVs were washed by centrifugation at 1500 rcf for 2 min and replacement of the supernatant with 40 μL of fresh outer phase, which was repeated twice. The resulting GUV suspensions (~40 μL) were used as stock solutions for the experiments described below.

Inner phase compositions: 10 mM acetate buffer, 150 mM NaCl, 200 mM sucrose. The pH was adjusted to 5 or 9 by adding small amounts of diluted HCl or NaOH. Where applicable, urease was added at a concentration of 3.5 mg/mL, calcein was added at 10 μM, dextran-fluorescein-tetramethylrhodamine (dx-FITC/TMR) was added at 0.1 mg/mL, DNA nanoswitch was added at 10 μM, nuclease (deoxyribonuclease I) was added at 1 mg/mL, acetylcholinesterase was added at 1.8 mg/mL. All components were added from concentrated stock solutions. Specific stock concentrations and added volumes for senders and receivers are summarized in the tables below.

Urease-based senders:

	Stock concentration	Added volume (μL) (for 25 μL of inner phase)	Final concentration
Buffer (acetate and NaCl)	50 and 750 mM	5	10 and 150 mM
Sucrose	1000 mM	5	200 mM
Urease	20 mg mL^{-1}	4.4	3.5 mg mL^{-1}
Water	-	10.6	-

DNA-nanoswitch receivers:

	Stock concentration	Added volume (µL) (for 25 µL of inner phase)	Final concentration
Buffer (acetate and NaCl)	50 and 750 mM	5	10 and 150 mM
Sucrose	1000 mM	5	200 mM
DNA	100 µM	2.5	10 µM
Water	-	12.5	-

Acetylcholinesterase-based senders:

	Stock concentration	Added volume (µL) (for 25 µL of inner phase)	Final Concentration
Buffer (acetate and NaCl)	50 and 750 mM	5	10 and 150 mM
Sucrose	1000 mM	5	200 mM
Enzyme	10 mg mL^{-1}	4.4	1.8 mg mL^{-1}
Water	-	10.6	-

Confocal laser scanning microscopy (CLSM) experiments

For microscopy experiments, GUVs were transferred into an 18-well µslide with a #1.5 glass coverslip bottom (Ibidi). First, the corresponding observation chamber was passivated by incubation with BSA (1 mg/mL) for at least 30 min and subsequently washed with Milli-Q water. Experiments corresponding to Figure S2 and S3 were performed on a Leica TCS SP8 using 488 nm and 552 nm laser lines, a HC PL APO CS2 20x/0.75 dry objective and a HyD detector. Calcein/fluorescein was excited at 488 nm and emission was collected at 495-535 nm; rhodamine was excited at 552 nm and emission was collected at 565-610 nm (512 x 512 resolution, sequential scanning). Experiments corresponding to Figure S4 and S17 were performed on a Leica TCS DMi8 using 488 and 532 nm laser lines, an ACS APO 40/1.15 oil objective and a PMT detector. For communication experiments, GUV imaging by CLSM was performed on a Leica TCS SP5X equipped with a white light laser, using a HCX PL APO CS 63x/1.20 water-immersion objective and HyD detectors. The image resolution was 1024 × 1024 pixels and scanning speed was 400 Hz. Bdp and Cy3-Cy5 were imaged using sequential scanning. Bdp-Chol was excited at 488 nm and emission was collected at 500-530 nm; DNA was excited at 530 nm and emission was collected at 550-620 nm (Cy3) and 640-715 nm (Cy5). In all cases, settings were fixed during the experiments to allow direct

comparison of the fluorescence intensities. Time-lapse imaging of microfluidic channels was performed a Leica TCS SP8 using a 552 nm laser lines (emission collected at 565-620 nm and 630-710 nm), a HC PL APO CS2 10x/0.40 dry objective (512 x 512 resolution) and a HyD detector. A Leica TCS DMi8 was employed for membrane experiments (Figure 2), using an ACS APO 40x/1.15 oil objective (512 x 512 resolution). Bdp-Chol was excited at 488 nm and emission was collected at 500-530 nm; DNA was excited at 532 nm and emission was collected at 550-620 nm (Cy3) and 640-715 nm (Cy5).

In all experiments, the indicated volume of GUV suspension(s) were pipetted from GUV stock solutions prepared as described above.

In a typical experiment, 6 µL of the corresponding GUV suspension(s) were added to the microscope chamber and the final volume was adjusted to 50 µL by addition of fresh outer phase (10 mM acetate buffer, 150 mM NaCl, 200 mM glucose). GUVs were let to settle for at least 5 min before input addition. For characterization of DNA-loaded receivers, 2 µL of 0.5% NH_3 was added to the chamber and incubated for 10 min followed by image acquisition. For communication experiments using urease-senders, samples were incubated for 3 hours in the presence or in the absence of urea (25 mM) before imaging. Urea stock solution was prepared at 1 M, and 1.25 µL of this stock were added to the microscope chamber to achieve 25 mM final concentration in the chamber.

For experiments using membrane duplex-functionalized GUVs (Figure 2), GUVs were prepared as described above. Then, 6 µL of the corresponding GUV suspension(s) were transferred to a microscope chamber and the final volume was adjusted to 50 µL by addition of fresh outer phase (10 mM acetate buffer, 150 mM NaCl, 200 mM glucose, pH 5). Then, 1 µL of ssDNA stock (stock concentration 100 µM, final concentration 2 µM) was added and the mixture was incubated for 10 min, followed by image acquisition. After confirming membrane recruitment of the ssDNA, 25 mM of urea was added (as described above), and the mixture was incubated for 1.5 h before image acquisition.

For experiments with the channel device (Figure 3), channels were loaded with 1 mg/mL BSA solution in Milli-Q and incubated for 1 h to passivate the surfaces. To remove the BSA solution, the channels were washed twice with Milli-Q and once with outer phase solution. Then, 50 µL of a solution containing receiver GUVs (10 µL of as-made stock plus 40 µL of outer phase solution) were loaded into the channel.

Subsequently, the reservoirs at the sides of the channel were loaded with 40 μL of outer phase. Then, 10 μl of sender GUV suspension was carefully deposited at the bottom of one of the reservoirs (outlet of the channel, see schematic in Figure 2), while 10 μL of outer phase were gently added in the other reservoir. The communication process was triggered by addition of 1 μL of urea stock solution (1 M) at the senders' location.

For communication experiments using acetylcholinesterase-senders (Figure S18), GUVs were first added to the microscope chamber (6 μL senders' stock solution and 6 μL of DNA-receivers) and completed to 50 μL by addition of fresh outer phase (10 mM acetate buffer, 150 mM NaCl, 200 mM glucose, adjusted to pH 9), and then alpha-hemolysin was added at a concentration of 10 μg/mL (addition of 1 μL of a 0.5 mg/mL stock) and let to incubate for at least 20 min. Then, acetylcholine was added at a final concentration of 5 mM.

For communication experiments using two sender populations (Figure 4), 6 μL of receiver GUVs were added in all cases, and the sender GUVs were added from their stock solutions to the microscope chamber in the following proportions: (a) 8 μL of acetylcholinesterase-GUVs and 8 μL of urease-GUVs, (b) 12 μL of acetylcholinesterase-GUVs and 4 μL of urease-GUVs, (c and d) 4 μL of acetylcholinesterase-GUVs and 12 μL of urease-GUVs. The volume in the chamber was completed to 50 μL with fresh outer phase at pH 9 and alpha-hemolysin was added at a concentration of 10 μg/mL (addition of 1 μL of a 0.5 mg/mL stock). Mixtures were let to incubate for 20 min. Then, substrates were simultaneously added at the indicated concentrations, and micrographs were acquired every 5 minutes. Specifically, a fuel stock solution containing 62.5 mM acetylcholine and 250 mM urea was prepared. 4.4 μL from this solution were added to each well to achieve a final concentration of 5 mM acetylcholine and 20 mM of urea, respectively. To achieve a final concentration of 15 mM acetylcholine and 10 mM of urea, a 4.4 μL aliquot was added from a stock solution containing 188 mM acetylcholine and 125 mM urea.

For spatiotemporal experiments in the microfluidic channels (Figure 5), 50 μL of a solution containing receiver GUVs (10 μL of as-made stock plus 40 μL of outer phase solution, pH 9) were loaded into the channel. Then, 20 μL of outer phase solution and 10 μL of either urease-GUVs, acetylcholinesterase-GUVs or additional outer phase were added in the reservoirs at the ends of the channel. The acetylcholinesterase-GUV reservoir was supplemented with 1 μL of alpha-hemolysin stock solution (0.5 mg/mL) and the

device was let to incubate for 20 min. Then, substrates were added at the senders' location at the corresponding concentration for each experiment and 15 µL of paraffin was added on top of the reservoirs to prevent water evaporation. Urea stock solution was prepared at 1 M, and 2.2 µL were added in the channel (containing 110 µL) to achieve a final concentration of 20 mM. Acetylcholine stock solution was prepared at a concentration of 500 mM, of which input volumes (1.1, 2.2 and 4.4 µL) were added to the channel to achieve the desired concentrations (5, 10 and 20 mM, respectively). Images of consecutive positions along the channel were acquired each 30 min.

For spatiotemporal experiments to induce triplex-duplex-triplex transitions (Figure 5F, S19), GUVs were set at pH 5 and loaded in the channel device as previously described with the two sender populations (urease-GUVs and acetylcholinesterase-GUVs) located either in opposite reservoirs (separated) or in the same reservoir (co-located). Acetylcholinesterase-GUVs were supplemented with 1 µL of alpha-hemolysin stock solution (0.5 mg/mL) and the device was let to incubate for 20 min. 5 mM of urea and 20 mM of acetylcholine were added from stock solutions (as described above) and 15 µL of paraffin was added on top of the reservoirs to prevent water evaporation. Receivers located near to the urease-GUVs reservoir (1.5-3 mm distance) were imaged in intervals of 1 h.

Image analysis

Micrographs were analyzed using Fiji (ImageJ) applying standard functions. To quantify the fluorescence output, individual GUVs were randomly selected as region of interest (ROI's) and manually tracked over subsequent frames while correcting for slight variations in position. Fluorescence intensities were extracted from the raw images (split channels) using the previously determined vesicle positions (ROI´s) and plotted. Although automatic selection could be performed using Fiji algorithms, in our case we found the manual tracking more efficient to discriminate between different types of GUVs in close proximity. The number (N) of analyzed GUVs in each experiment (indicated in figure captions) was chosen for convenience (although samples contained a larger number of GUVs) as it gave a representative overview of the sample and increasing the number of analyzed GUVs did not have a significant effect on the mean and standard deviation. To extract membrane intensity (Figure 2), two intensity line profiles were plotted for each GUV and the average maximum was extracted.

Supplementary Figures

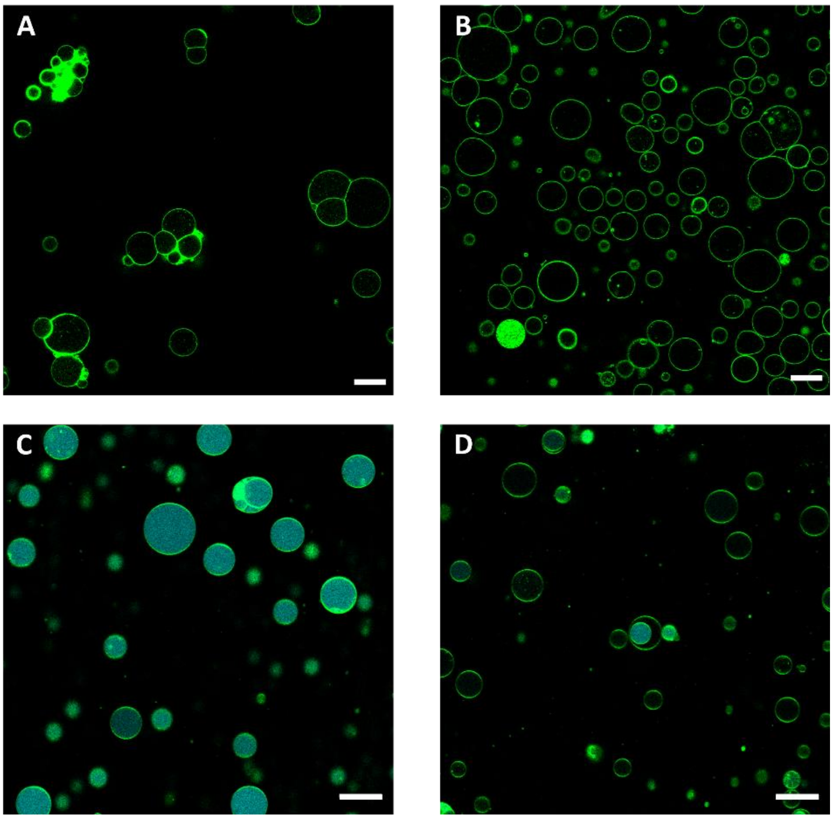

Figure S2. Study of the preparation of urease-loaded sender GUVs. **A)** Micrograph of GUVs containing 0% of pegylated lipid. GUVs are indeed formed but they appear clustered. **B)** Micrograph of GUVs containing 1% of pegylated lipid. GUV clustering is prevented and the yield increases. Bodipy-cholesterol was used as membrane marker. C-D) Loading of molecular cargo and integrity of the GUV membrane. **C)** GUVs labelled with membrane marker (green, DOPE-LRB) entrap molecular cargo (cyan, calcein) added in the inner phase during the preparation process. **D)** Oil-free membrane and unilamellarity are confirmed by addition α-hemolysin to the external medium, which induces the release of calcein and thus the decrease of lumen fluorescence. Alpha-hemolysin, as a pore-creating protein, assembles in the membrane and allows the diffusion of small charged molecules such as calcein, which confirms membrane functionality. Scale bars represent 20 µm.

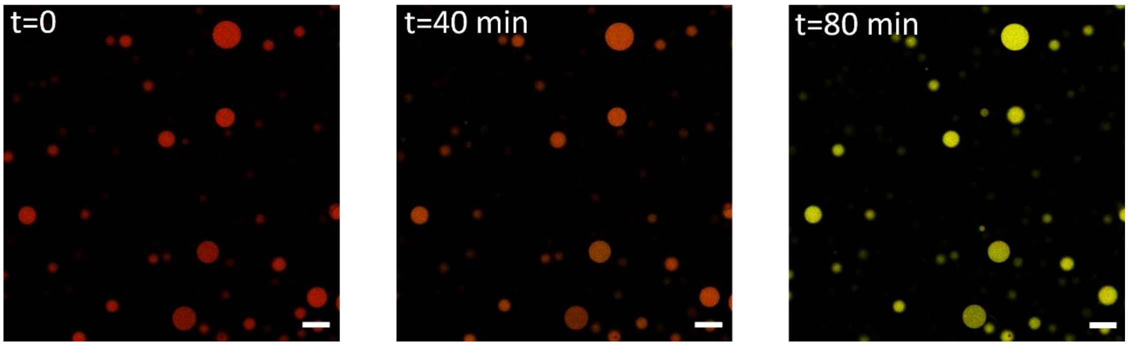

Figure S3. Monitoring of urea conversion in urease-loaded GUVs, co-loaded with dx-FITC-TMR (pH sensor). Micrographs at different times upon addition of urea (25 mM), showing overlaid channels corresponding to rhodamine (red) and fluorescein (green) – resulting in yellow as an indication of lumen basification (due to the conversion of urea to ammonia). The change in color correlated with an increase in pH from 5 (at t = 0 min) to 9 (at t = 80 min). Scale bar represents 20 µm.

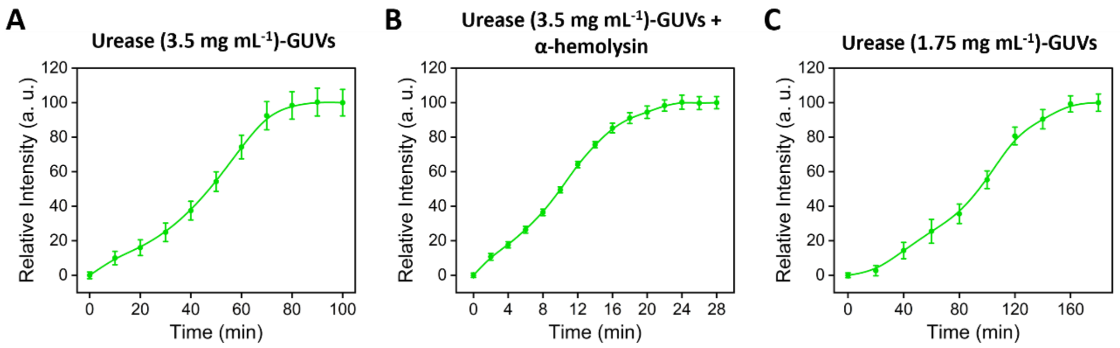

Figure S4. Kinetics of lumen basification (due to the conversion of urea to ammonia) in urease-loaded GUVs under different conditions, showing the relative increase in FITC/TMR intensity (pH sensor) as a function of time. Urea was added at a concentration of 25 mM. The change in intensity correlates with an increase in pH from 5 (at t=0) to 9 (maximum intensity). **A)** GUVs with 3.5 mg mL^{-1} of urease showed conversion, thus indicating the permeation of urea through the lipid membrane. **B)** Upon treatment with α-hemolysin (pore-forming protein, 10 µg mL^{-1}), the high permeability of the lipid membrane results in faster kinetics (compared to A). **C)** Conversion in GUVs loaded with 1.75 mg mL^{-1} of urease. The rate of ammonia production (lumen basification) is slowed down (compared to A) when the amount of urease (incorporated in the GUV lumen during the preparation process) is reduced.

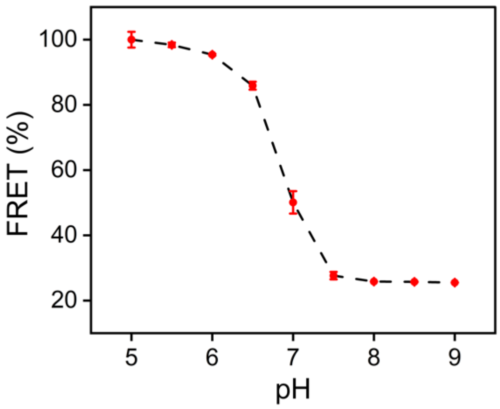

Figure S5. Relative FRET emission corresponding to the triplex-to-duplex transition of the DNA nanoswitch as a function of pH. Upon excitation at 530 nm, the maxima of fluorescence at 563 nm (Cy3) and at 660 nm (Cy5) were registered. FRET (%) was calculated as $(F_{Cy5}/F_{Cy3})_i/(F_{Cy5}/F_{Cy3})_{pH\text{-}5} \times 100$. DNA nanoswitch (500 nM) was added in PBS (1X) at the corresponding pH. Error bars represent the s. d. from two independent measurements.

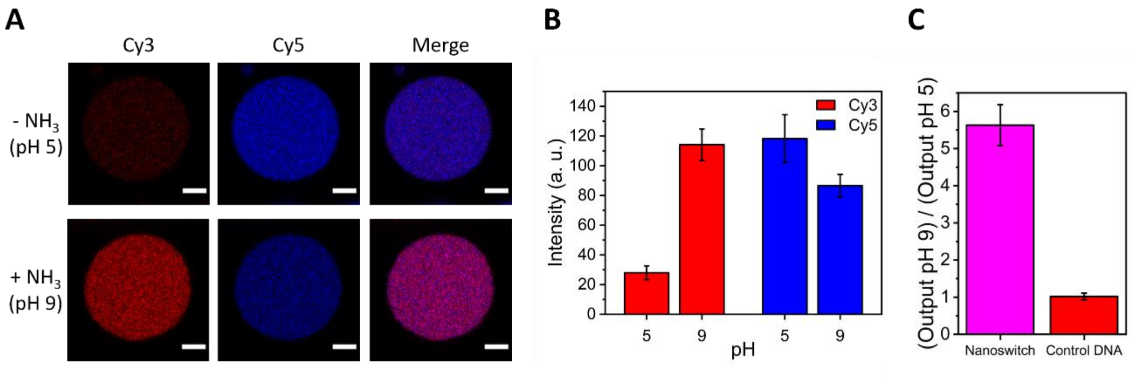

Figure S6. Characterization of DNA-nanoswitch-loaded receiver GUVs. **A)** Micrographs showing from left to right the Cy3 channel, Cy5 (FRET acceptor) channel and merge upon excitation of Cy3 (FRET donor); in the absence (top, pH 5) and presence (bottom, pH 9) of ammonia (10 mM) added to the external medium. Scale bars represent 5 µm. **B)** Quantification of Cy3 and Cy5 emission intensity from GUVs in the absence (pH 5) and upon addition of ammonia (pH 9). **C)** Output (Cy3-intensity / Cy5-intensity) ratio between pH 9 and pH 5 from GUVs containing DNA nanoswitch and control DNA, respectively; where 1 represents no responsiveness. Data plotted as mean ± s.d. from multiple GUVs ($N \geq 35$).

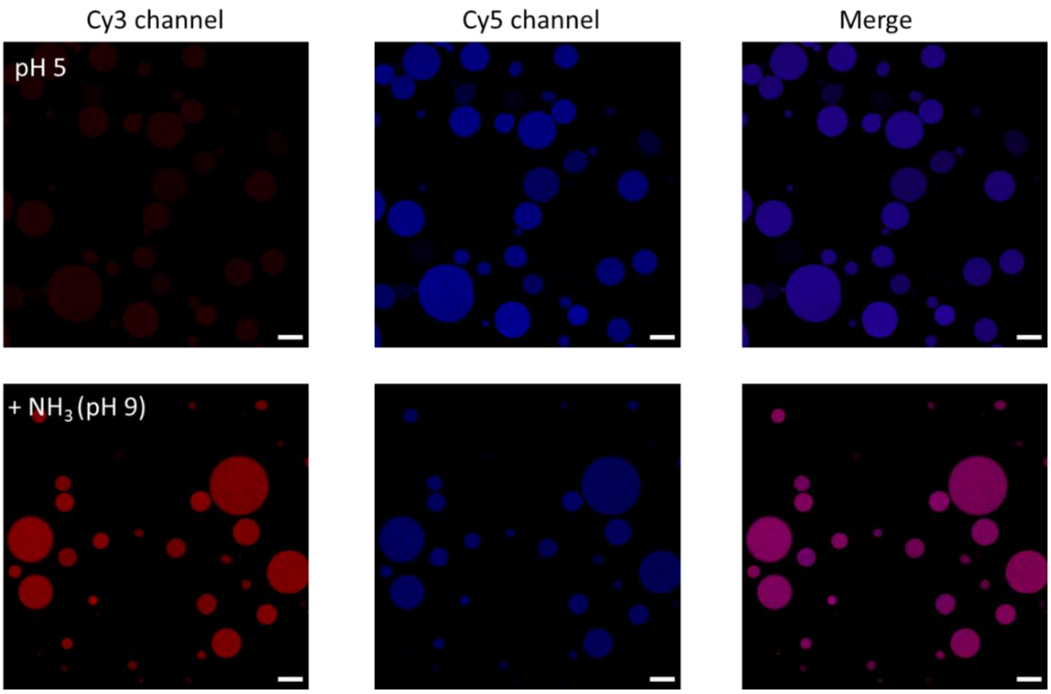

Figure S7. Representative full field microscopy views of DNA-loaded receiver GUVs in the absence (pH 5, top) and presence of ammonia (10 mM, pH 9, bottom).

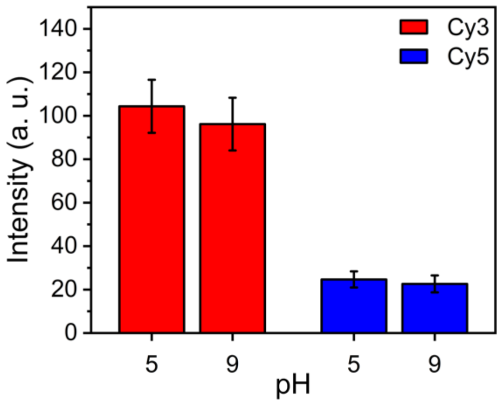

Figure S8. Quantification of Cy3 and Cy5 fluorescence intensity in GUVs loaded with control DNA (Cy3 and Cy5-labelled random sequences) in the absence (pH 5) and presence of ammonia (pH 9) in the medium. Cy3 and Cy5 fluorescence intensities do not show a significant variation with pH. Representative micrographs are showed in Figure SI-7. Error bars represent the s. d. from multiple GUVs ($N \geq 100$ GUVs).

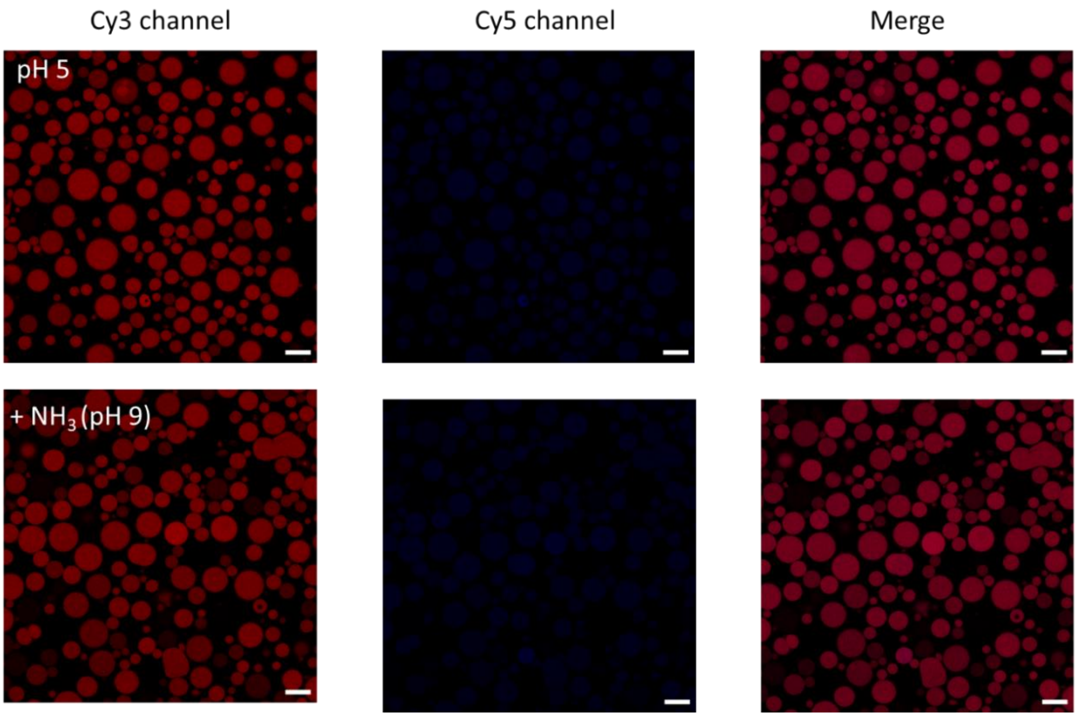

Figure S9. GUVs loaded with control DNA (Cy3 and Cy5-labelled random sequences) do not respond to addition of ammonia (pH basification). Representative full field views of control-DNA GUVs in the absence (pH 5, top) and in the presence of ammonia in the medium (10 mM, pH 9, bottom).

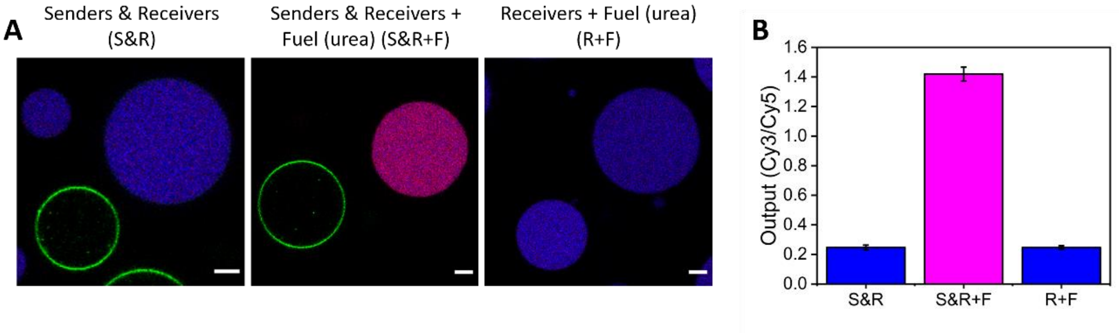

Figure S10. A) Micrographs of chemical communication experiments between senders (urease-loaded, green membrane labelling) and receiver GUVs (DNA-loaded) in close proximity. (left) In the absence of urea (fuel), the entrapped DNA nanostructures remain in the triplex state (blue); (middle) with input of urea, communication leads to the switching of the DNA nanostructure to the duplex conformation (pink); (right) in the absence of senders, there is no response. Scale bars represent 5 μm. **B**) Output (Cy3-Cy5 emission ratio) quantification for the different conditions: sender-receiver consortium in the absence of urea (S&R), sender-receiver consortium in the presence of urea (S&R+F), and receiver population in the presence of urea (R+F). Data plotted as mean ± s.d. (N ≥ 20 GUVs).

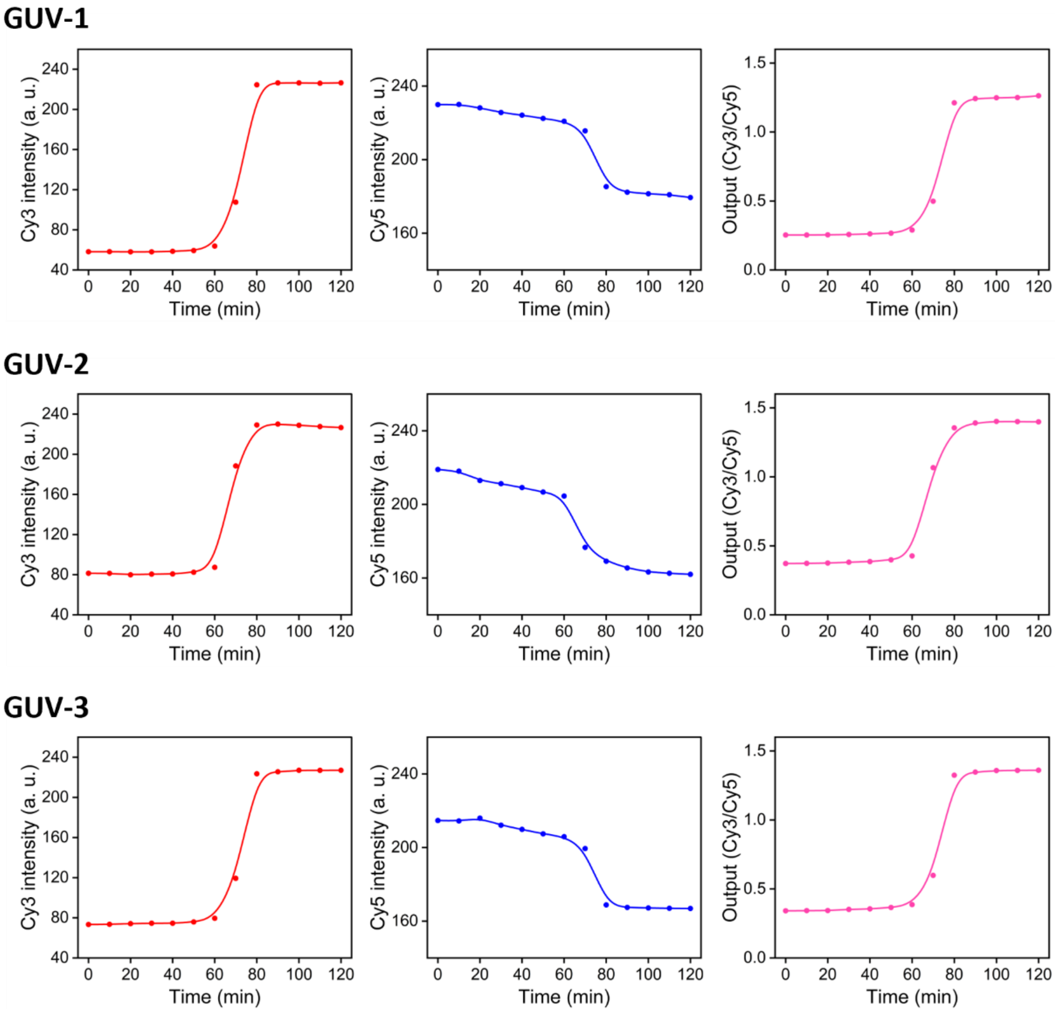

Figure S11. Time-dependent fluorescence profiles of three different receiver GUVs upon signaling from sender vesicles in the presence of urea. Changes in Cy3 and Cy5 emission demonstrate conformational change of the DNA nanostructure.

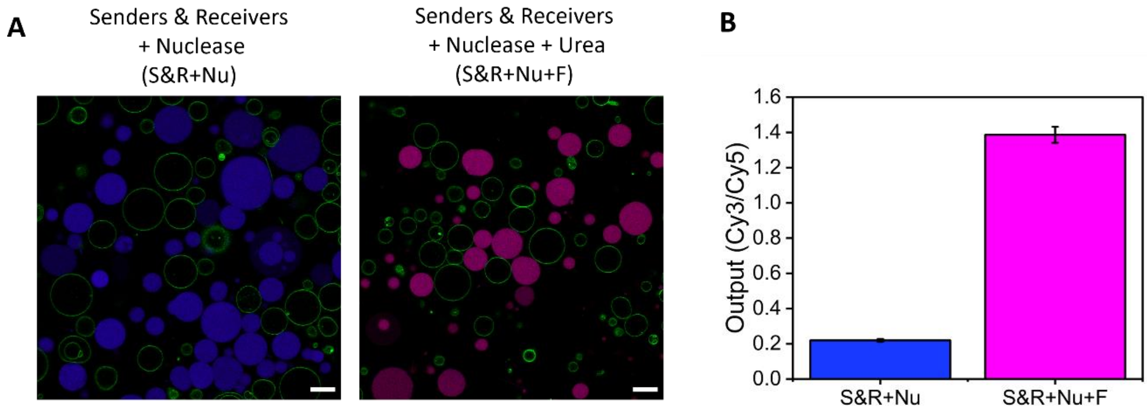

Figure S12. Protection against degradation by nuclease. **A)** Micrographs of sender and receiver GUVs in the presence of nuclease (1 mg/mL) in the external medium in the absence or presence of urea (25 mM). **B)** Output (Cy3-Cy5 emission ratio) from sender/receiver consortium upon addition of nuclease to the external medium in the presence and absence of urea. Compartmentalization offers protection to the encapsulated molecular transceivers/receivers and communication is not affected by the presence of potential degrading agents in the external medium. Scale bars represent 20 µm. Error bars represent the s. d. from multiple GUVs ($N \geq 25$ GUVs).

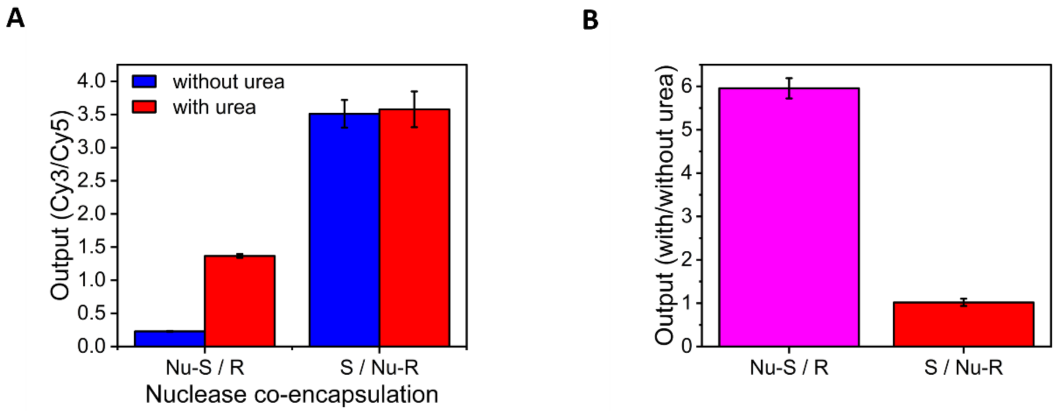

Figure S13. Chemical communication experiments between sender and receiver GUVs containing nuclease. **A)** Output (Cy3-Cy5 emission ratio) quantification for different consortia in the absence and presence of urea: with nuclease incorporated in sender GUVs (Nu-S / R), and with nuclease incorporated in receiver GUVs (S / Nu-R). **B)** Relative response to input of urea. The communication system is functional when nuclease is incorporated in sender GUVs, whereas the consortium becomes unresponsive when nuclease is incorporated in receivers (output ratio=1). Data plotted as mean ± s.d. (N ≥ 18 GUVs).

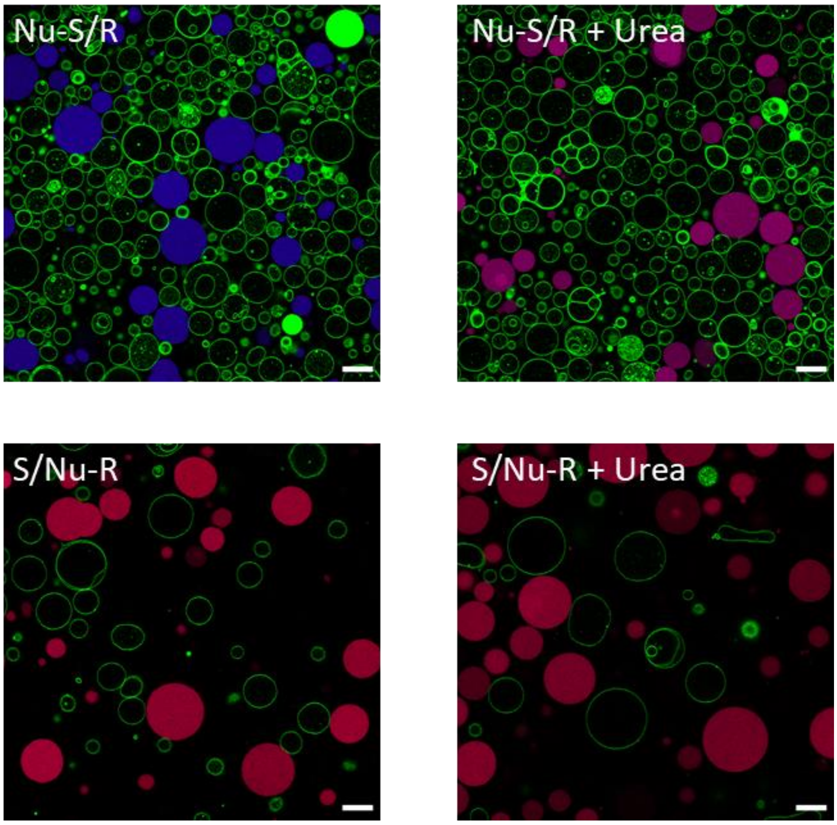

Figure S14. Micrographs corresponding to the plot depicted in Figure S11. Sender-receiver consortia incorporating nuclease enzyme in sender GUVs (Nu-S/R) retain their communication capability and are able to read the presence of urea in the medium, which induces DNA nanostructure triplex(blue)-to-duplex(pink) transition in receivers. In contrast, sender-receiver consortia with nuclease enzyme in receiver GUVs (S/Nu-R) are not able to respond to urea — the DNA nanostructure is degraded by the co-compartmentalization with nuclease enzyme. Scale bars represent 20 µm.

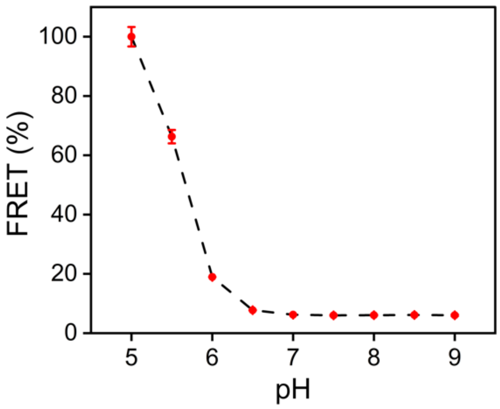

Figure S15. Relative FRET emission as a function of pH for the DNA duplex and ssDNA able to assemble by Hoogsteen interactions. Upon excitation at 530 nm, the maxima of fluorescence at 563 nm (Cy3) and at 660 nm (Cy5) were registered. FRET (%) was calculated as $(F_{Cy5}/F_{Cy3})_i/(F_{Cy5}/F_{Cy3})_{pH\text{-}5} \times 100$. Both sequences were added at 500 nM in PBS (1X) at the corresponding pH. Error bars represent the s. d. from two independent measurements.

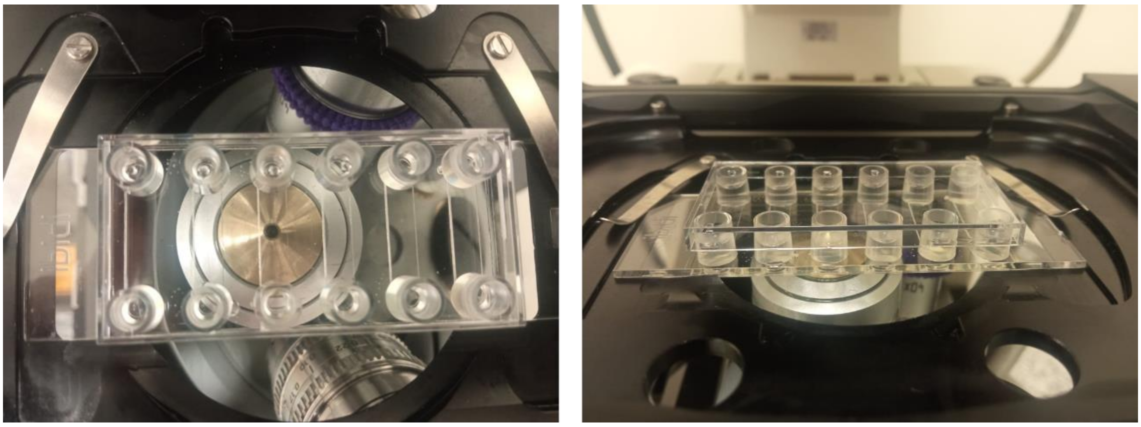

Figure S16. Photographs of the microfluidic channels employed in our study (mounted on the confocal microscope). The commercial device (from Ibidi) consists of a microscope slide (glass bottom) with six parallel channels with the following dimensions: length = 17 mm, width = 3.8 mm, height = 0.54 mm; channel volume = 40μL, volume per reservoir = 60 μL.

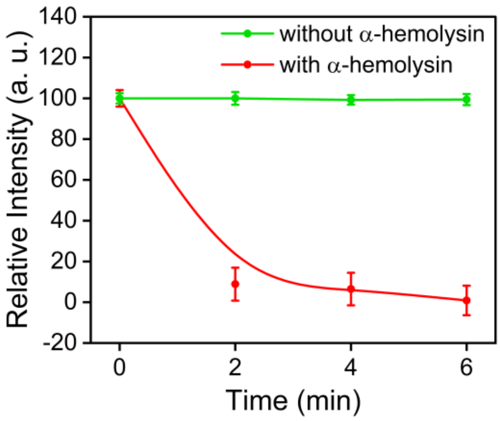

Figure S17. Monitoring of acetylcholine conversion in acetylcholinesterase-loaded GUVs, co-loaded with dx-FITC-TMR (pH sensor), treated without and with α-hemolysin (10 µg mL^{-1}). Acetylcholine concentration was 5 mM. Maximum intensity corresponds to GUVs at pH 9, and minimum intensity corresponds to acidification to pH 5. As observed, addition of α-hemolysin facilitates the permeation of acetylcholine, resulting in fast production of acetic acid.

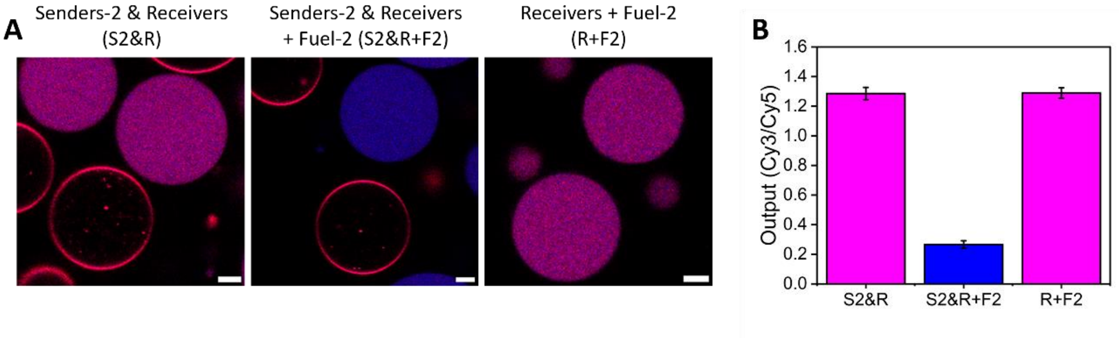

Figure S18. A) Micrographs of chemical communication experiments between senders-2 (acetylcholinesterase-loaded, red membrane labelling) and receiver GUVs (DNA-loaded) in close proximity. (left) In the absence of acetylcholine (fuel-2, 5 mM), the entrapped DNA nanostructure remains in the triplex state (pink); (middle) with input of urea, communication leads to the switching of the DNA nanostructure to the duplex conformation (blue); (right) in the absence of senders, there is no response. Scale bars represent 5 μm. **B**) Output (Cy3-Cy5 emission ratio) quantification for the different conditions: sender-receiver consortium in the absence of acetylcholine (S2&R), sender-receiver consortium in the presence of acetylcholine (S2&R+F2), and receiver population in the presence of acetylcholine (R+F2). Data plotted as mean ± s.d. (N = 10 GUVs).

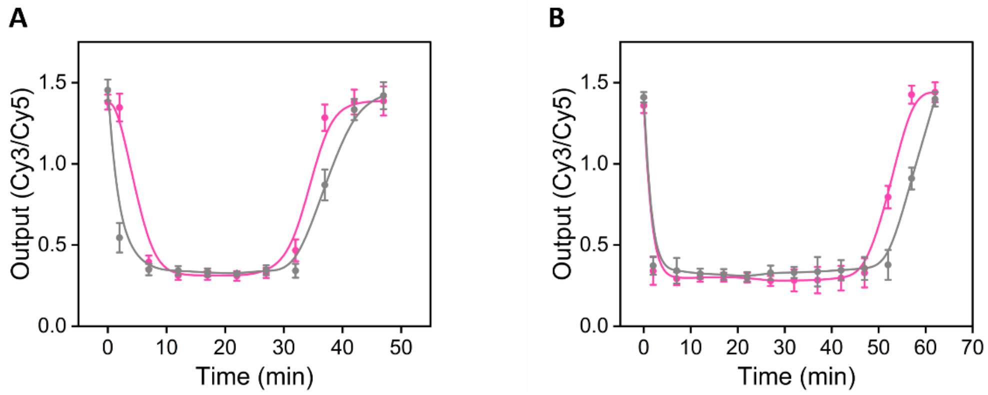

Figure S19. Comparative time-dependent output from two independent repeats (pink and grey lines, respectively) of dynamic reorganization of DNA nanostructures in consortia with two antagonistic sender populations. A) Equal proportion of sender population-1 (urease-loaded, green membrane in Figure 4) and sender population-2 (acetylcholinesterase-loaded, red membrane in Figure 4). B) Major proportion of population-2 (acetylcholinesterase-loaded) *vs*. population-1 (urease-loaded). Data plotted as mean ± s.d. (N = 14 GUVs). As observed, similar trends were obtained in independent repeats (pink and grey).

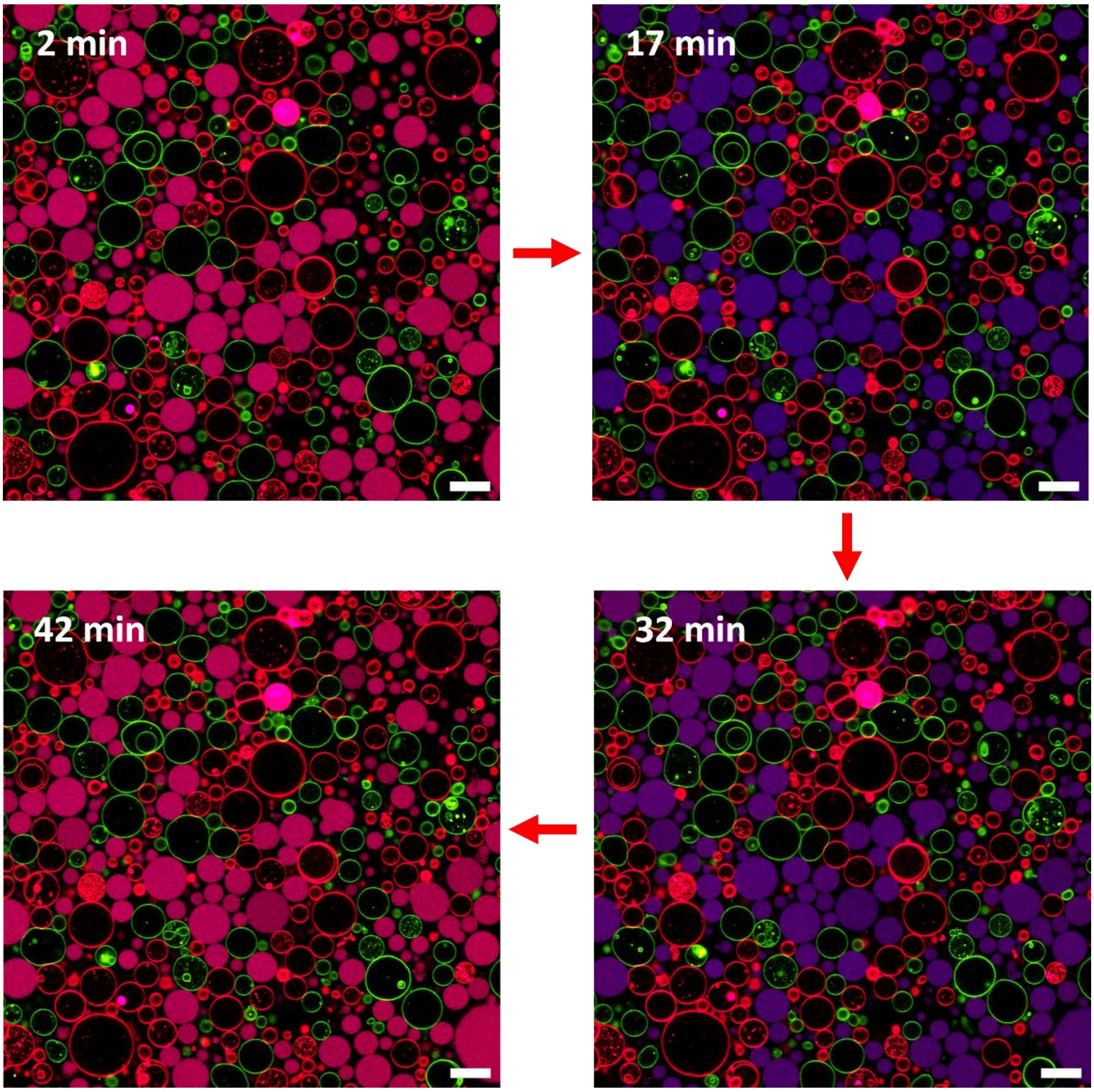

Figure S20. Communication in artificial cell consortia with two antagonistic sender populations drive dynamic out-of-equilibrium reorganization of DNA nanostructures in receivers. Illustrative micrographs at different times of a community composed of equal amounts of sender population-1 (green-membrane, urease-loaded) and sender population-2 (red-membrane, acetylcholinesterase-loaded). Scale bars represents 20 µm.

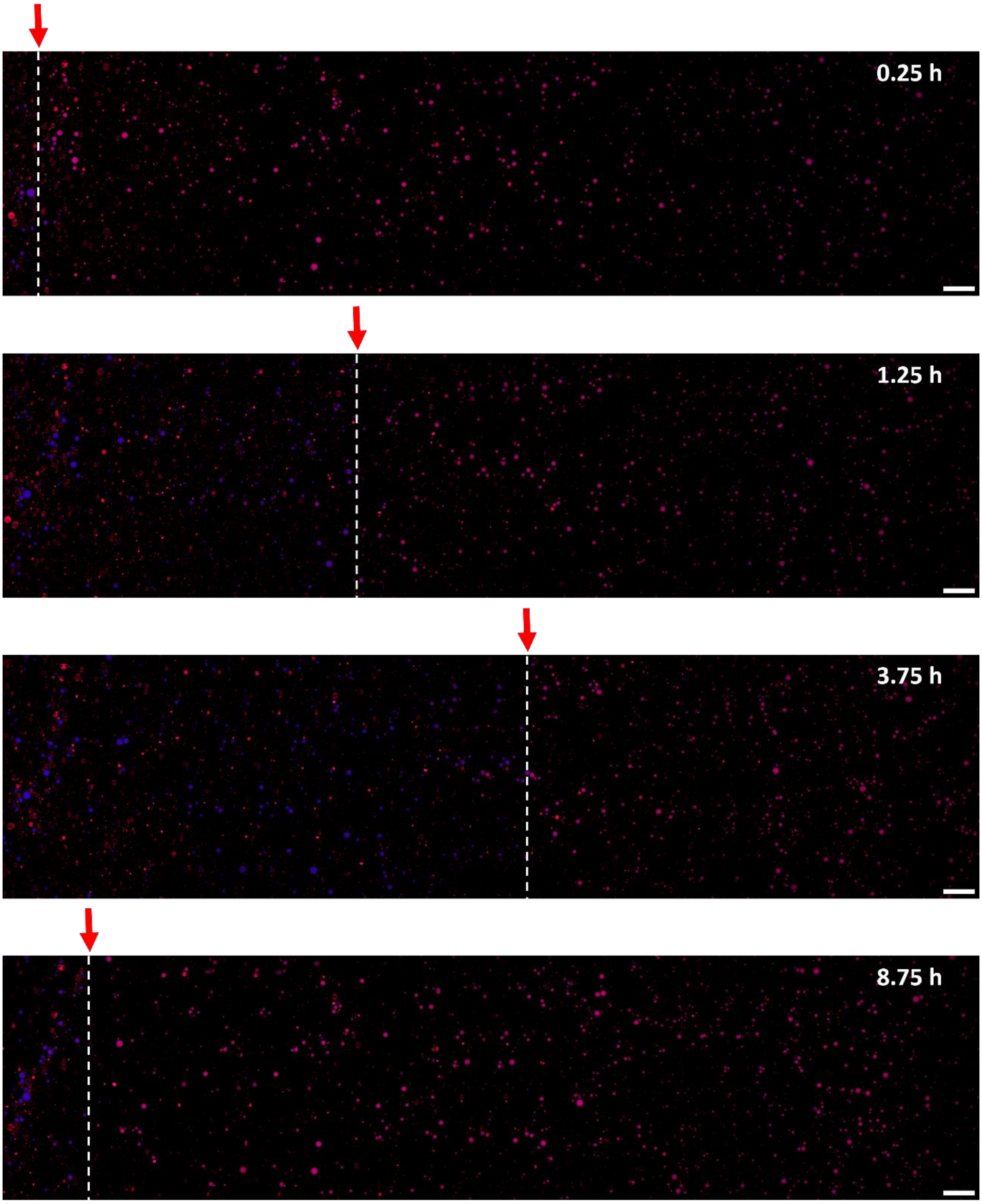

Figure S21. Spatiotemporal communication drives transient activation of receivers. Reconstructed micrographs at different times of the microfluidic channel containing receivers when using 5 mM of ACh and 20 mM of urea, upon signaling from the two antagonistic sender populations (each located at opposite ends in the microfluidic device, not shown in the image). The advance and recession of the signaling front can be appreciated, as indicated by the arrow and white dashed line. Scale bars represent 200 μm.

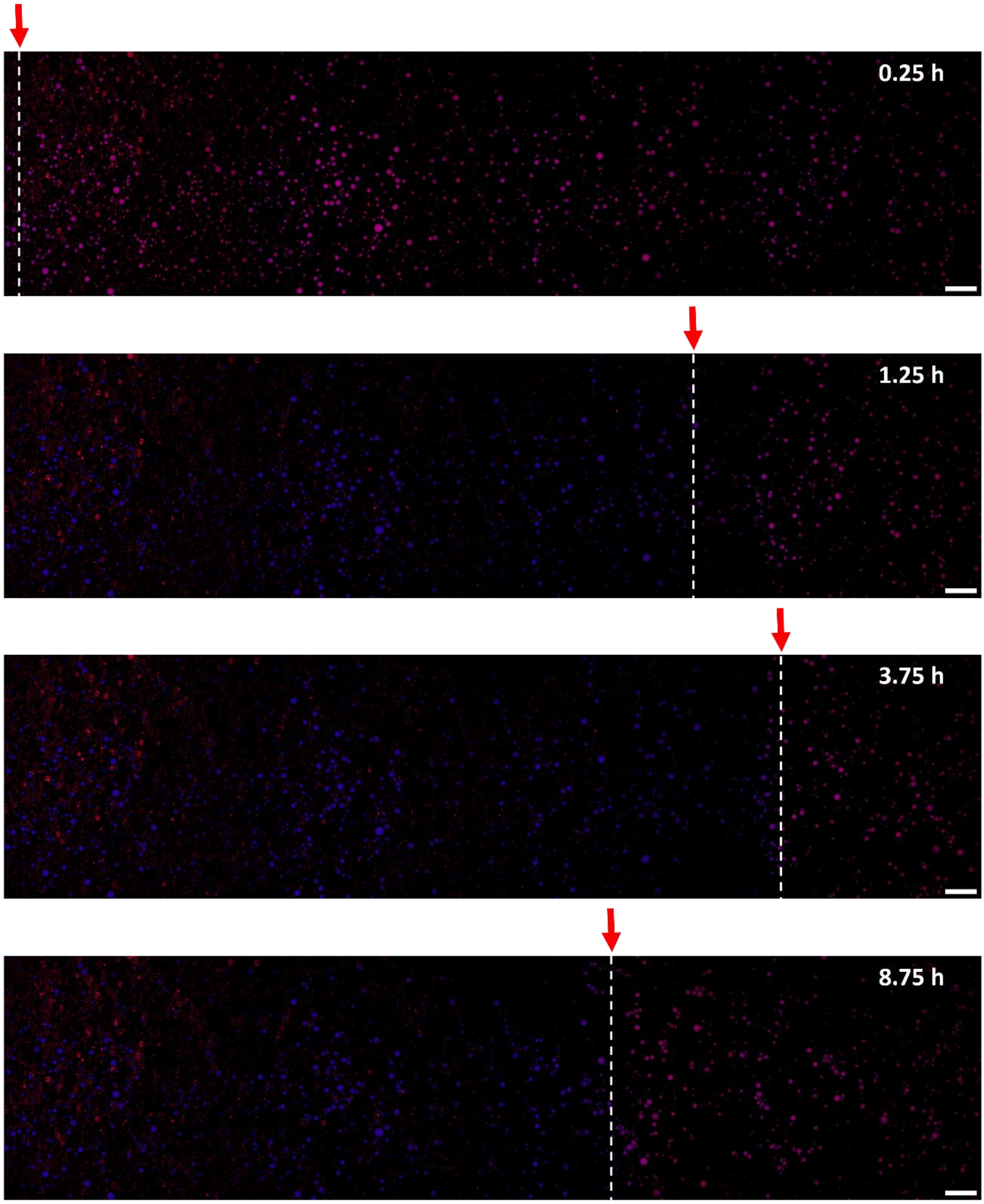

Figure S22. Spatiotemporal communication drives transient activation of receivers. Reconstructed micrographs at different times of the microfluidic channel containing receivers when using 20 mM of ACh and 20 mM of urea, upon signaling from the two antagonistic sender populations (each located at opposite ends in the microfluidic device, not shown in the image). The advance and recession of the signaling front can be appreciated, as indicated by the arrow and white dashed line. Scale bars represent 200 µm.

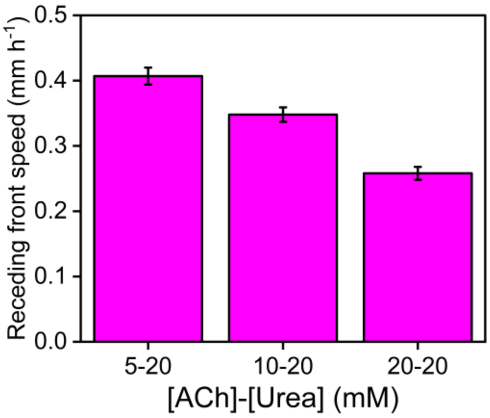

Figure S23. Speed of the receding front (receivers switched to the triplex state) under different substrate concentrations, as extracted by linear fitting of the distance vs. time plots (Figure 5).

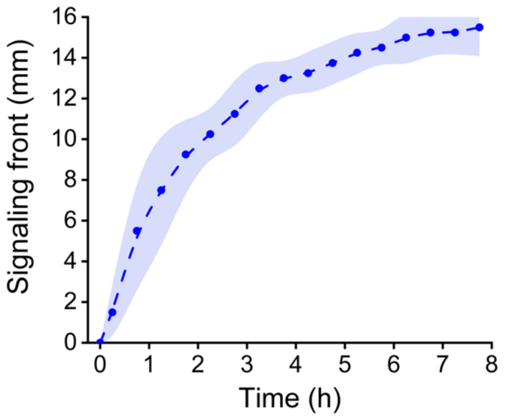

Figure S24. Signaling front (furthest located receiver with DNA in the duplex state) as a function of their distance to the acid-producing acetylcholinesterase-senders, when no urease-senders were used.

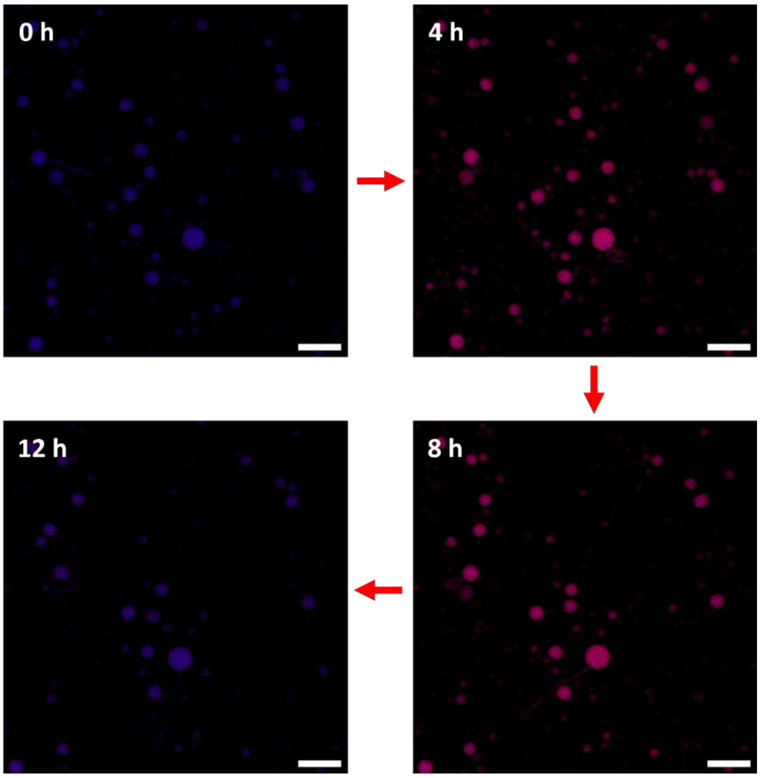

Figure S25. Micrographs at different times of receivers undergoing triplex-duplex-triplex transition upon signaling from the two antagonistic sender populations (each located in opposite ends in the microfluidic device). Receivers were imaged at 1.5-3 mm distance from the urease population. The system was initially set at pH 5, and 5 mM of urea and 20 mM of acetylcholine were simultaneously added. Scale bars represent 100 µm.

Supplementary References

1. T. Patino, A. Porchetta, A. Jannasch, A. Lladó, T. Stumpp, E. Schäffer, F. Ricci and S. Sánchez. Self-Sensing Enzyme-Powered Micromotors Equipped with pH-Responsive DNA Nanoswitches. *Nano Lett.*, 2019, **19**, 3440–3447.
2. M. E. Fornace, J. Huang, C. T. Newman, N. J. Porubsky, M. B. Pierce, N. A. Pierce. NUPACK: analysis and design of nucleic acid structures, devices, and systems. *ChemRxiv*, **2022**.
3. B. C. Buddingh', A. Llopis-Lorente, L. K. E. A. Abdelmohsen and J. C. M. van Hest. Dynamic spatial and structural organization in artificial cells regulates signal processing by protein scaffolding. *Chem. Sci.*, 2021, **11**, 12829–12834.
