# Supplementary material for: Spatiotemporal Communication in Artificial Cell Consortia for Dynamic Control of DNA Nanostructures
Source: ACS Cent Sci. 2024 Jul 22;10(8):1619–28. doi: 10.1021/acscentsci.4c00702 (PMC11363350; doi:10.1021/acscentsci.4c00702)
Supplement: Supplementary file 5 — oc4c00702_si_005.pdf [file oc4c00702_si_005.pdf]

oc-2024-007026.R1

Name: Peer Review Information for "Spatiotemporal Communication in Artificial Cell Consortia for Dynamic Control of DNA Nanostructures"

First Round of Reviewer Comments

Reviewer: 1

Comments to the Author

In this manuscript, the authors present an innovative approach to spatiotemporal communication between cellular-scale entities, functioning as both senders and receivers. They effectively utilize transmitted signals to induce conformational changes within compartmentalized DNA structures, demonstrating transient, distance-dependent activation facilitated by strategically positioned sender populations. While the results and conclusions appear robust and convincing, a major revision is necessary to address the following concerns:

1. The permeability of the lipid membrane to acetylcholine and urea, which are critical to signal communication, should be thoroughly investigated. Detailed descriptions of the methods for preparing and characterizing the GUVs, including explicit concentrations and conditions for encapsulating enzymes and DNA, must be provided in the experimental section.

2. Add a schematic to Figure 2 to clearly convey the experimental design and processes described in this work.

3. Since the microfluidic channel is crucial for spatiotemporal signal communications, please include a photograph of the channels in the supplementary information (SI) and provide detailed parameter information in the experimental section.

4. To emphasize "Spatiotemporal Communication," please supplement Figure 1 with time-dependent fluorescence imaging for a single vesicle at a fixed position, demonstrating the pH-dependent DNA conformation change in receiver vesicles.

5. Cite relevant literatures associated with protocell signal communications or oscillating pH regulation, specifically: Chem. Sci., 2019, 10, 9446-9453; Small Methods, 2023, 7, e2300042; Chem 2024, 10, 600–614.

Reviewer: 2

Comments to the Author

This manuscript aims to advance the communication between artificial cells. They present an overall interesting idea but the manuscript is very condensed and full of fancy sounding terms, which make the reading of the manuscript difficult. The introduction would also benefit from some more depth of such an active field. Overall, the manuscript could become publishable in this journal after major revisions. In particularly, the authors have provide stronger argumentation while their results that are largely based on the diffusion of small molecules are illustration control and communication.

Comments:

- The text that goes with Figure 2 is too short and it is difficult to understand what the authors want to illustrate and why. Where is the SSDNA? Outside of the GUV?

- Is it really fair to call the results in Figure 3 spatiotemporal communication since it is simple small molecule diffusion?

- In Figure 4, does N=14 mean that 14 GUVs were analyzed? That seems a very small set since the authors have no problem to create large populations. Are there independent repeats to illustrate the variability in encapsulation? Can the authors control their system by controlling how much material they encapsulate?

- ‘Such transient spatiotemporal activation patterns are reminiscent of cellular signaling in multicellular organisms, where molecules such as hormones and morphogens display dynamic concentration gradients that drive different biological processes in receivers.’ is in my option a very strong overstatement to what the authors show.

- The signaling front should be included in Fig 5a. Alternatively, all the sub-figs in 5 should have a illustration. The text is very condensed, which makes it difficult to understand what is actually happening. I am sure I understand how this experiments show transient spatiotemporal activation patterns

- The manuscript would also benefit from a more elaborate statement on how this would be eventually useful for creating avenues for engineering

Author's Response to Peer Review Comments:

Dear Editor:

Thank you very much for your e-mail regarding the manuscript oc-2024-007026 entitled "Spatiotemporal Communication in Artificial Cell Consortia for Dynamic Control of DNA Nanostructures".

We would like to thank the reviewers for their constructive comments. Please find below a point-by-point response to the referees' comments including a description of the changes and additional experiments we have included in this revised version of the manuscript. We have highlighted these changes in the revised manuscript and Supporting Information.

We hope that these additions to the manuscript make it suitable for publication by ACS Central Science.

Yours faithfully,

Prof. Loai K. E. A. Abdelmohsen

Manuscript ID: oc-2024-007026

Dear Prof. Kirk S. Schanze:

Thank you very much for your e-mail regarding the manuscript oc-2024-007026 entitled "**Spatiotemporal Communication in Artificial Cell Consortia for Dynamic Control of DNA Nanostructures**".

We would like to thank the reviewers for their constructive comments. Please find below a point-by-point response to the referees' comments including a description of the changes and additional experiments we have included in this revised version of the manuscript. We have highlighted these changes in the revised manuscript and Supporting Information.

We hope that these additions to the manuscript make it suitable for publication by ACS Central Science.

Yours faithfully,

Prof. Loai K. E. A. Abdelmohsen

Editorial comments:

Formatting needs:

SYNOPSIS MISSING: The synopsis should be no more than 200 characters (including spaces) and should reasonably correlate with the TOC graphic. The synopsis is intended to explain the importance of the article to a broader readership across the sciences. Please place your synopsis in the manuscript file after the TOC graphic, and label it as "Synopsis."

The following synopsis has been added as requested: "*Consortia of sender and receiver artificial cell mimics are designed to orchestrate dynamic reconfiguration of DNA nanostructures upon transformation of chemical fuels, with spatiotemporal control*."

SI PARAGRAPH: If the manuscript is accompanied by any supporting information for publication, a brief description of the supplementary material is required in the manuscript. The appropriate format is: Supporting Information. Brief statement in non-sentence format listing the contents of the material supplied as Supporting Information.

A brief description of the Supporting Information has been added to the manuscript as follows: "***Supplementary Information***

Materials and methods, Figures S1 to S25, supplementary references (PDF).

Video S1_Urease-senders and DNA-receivers (AVI).

Video S2_ Acetylcholinesterase-senders and DNA-receivers (AVI).

Video S3_Three population consortium with equal proportion of senders (AVI).”

Reviewer 1:

In this manuscript, the authors present an innovative approach to spatiotemporal communication between cellular-scale entities, functioning as both senders and receivers. They effectively utilize transmitted signals to induce conformational changes within compartmentalized DNA structures, demonstrating transient, distance-dependent activation facilitated by strategically positioned sender populations. While the results and conclusions appear robust and convincing, a major revision is necessary to address the following concerns:

We thank the reviewer for their positive assessment and clear summary of our work.

1. The permeability of the lipid membrane to acetylcholine and urea, which are critical to signal communication, should be thoroughly investigated. Detailed descriptions of the methods for preparing and characterizing the GUVs, including explicit concentrations and conditions for encapsulating enzymes and DNA, must be provided in the experimental section.

Regarding the permeability of the lipid membrane to urea and acetylcholine, we would like to point out that:

- Urea molecules had previously been reported to permeate through lipid membranes (*Chem Sci.* 2020, *11*, 3228). Accordingly, we performed the experiments described in Figures 1, 2 and 3 (involving urease-senders and DNA-receivers) considering urea as a permeable substrate.
- Following the reviewer’s comment, we have conducted additional experiments in which the permeability of the lipid membrane to urea was evaluated by measuring the kinetics of urea transformation (lumen basification) in urease-loaded GUVs (Figure S4). We compared GUVs with α-hemolysin pores (highly permeable membrane to allow the direct pass-through of chemicals) and GUVs without α-hemolysin (semipermeable membrane). Indeed, in both cases an increase in pH was observed in the GUV lumen – thus, indicating the permeation of urea through the lipid membrane - in line with

previous studies. Moreover, the addition of α-hemolysin (enabling direct diffusion of urea to the lumen) was observed to result in faster kinetics, which indicates that the rate of urea transformation is partially slowed down by the GUV membrane (in the absence of α-hemolysin). This effect may potentially be leveraged to modulate enzymatically-driven processes based on tuning membrane permeability.

- Acetylcholine had previously been reported to need membrane carriers to permeate through lipid membranes (*Nature* 2022, *603*, 637). Accordingly, experiments involving acetylcholine were performed using GUVs with α-hemolysin to ensure access to the GUV lumen.
- Following the reviewer's comments, we carried out additional experiments to check the kinetics of acetylcholine transformation by acetylcholinesterase-loaded GUVs upon incubation (or not) with pore-forming α-hemolysin. As expected, acetylcholine-transformation was facilitated in α-hemolysin-treated GUVs (Figure S17).

These points and additional experiments have been included in the revised version of the manuscript.

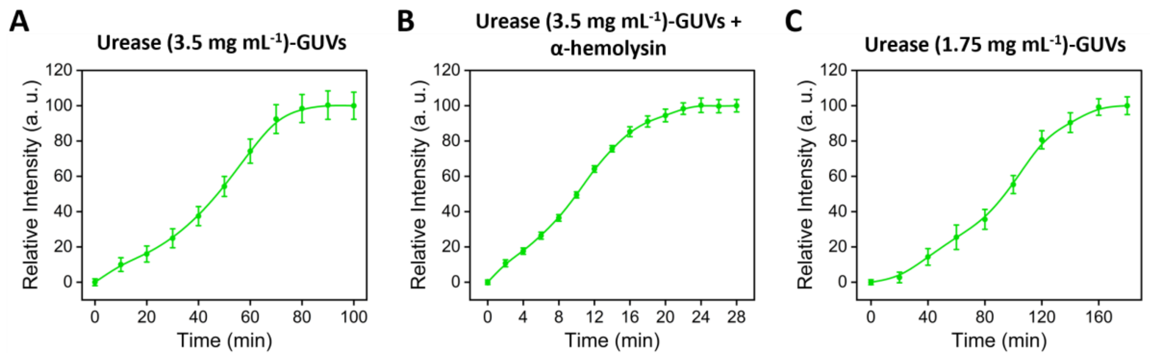

Figure S4. Kinetics of lumen basification (due to the conversion of urea to ammonia) in urease-loaded GUVs under different conditions, showing the relative increase in dx-FITC/TMR intensity (pH sensor) as a function of time. Urea was added at a concentration of 25 mM. The change in intensity correlates with an increase in pH from 5 (at t=0) to 9 (maximum intensity). **A)** GUVs with 3.5 mg mL^{-1} of urease showed conversion, thus indicating the permeation of urea through the lipid membrane. **B)** Upon treatment with α-hemolysin (pore-forming protein, 10 µg mL^{-1}), the high permeability of the lipid membrane results in faster kinetics (compared to A). **C)** Conversion in GUVs loaded with 1.75 mg mL^{-1} of urease. The rate of ammonia production (lumen basification) is slowed down (compared to A) when the amount of urease (incorporated in the GUV lumen during the preparation process) is reduced.

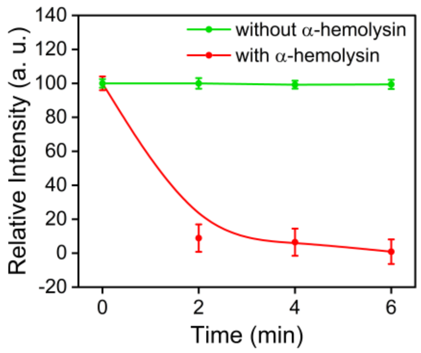

Figure S17. Monitoring of acetylcholine conversion in acetylcholinesterase-loaded GUVs, co-loaded with dx-FITC/TMR (pH sensor), treated without and with α-hemolysin (10 μg mL^{-1}). Acetylcholine concentration was 5 mM. Maximum intensity corresponds to GUVs at pH 9, and minimum intensity corresponds to acidification to pH 5. As observed, addition of α-hemolysin facilitates the permeation of acetylcholine, resulting in fast production of acetic acid.

Finally, as suggested by the reviewer, we have revised the experimental section and supplemented further details including explicit concentrations and conditions for encapsulating enzymes and DNA as exemplified in the tables below. Concentrations of substrate stock solutions and added volumes have also been added. See the tables below.

Urease-based senders:

	Stock concentration	Added volume (μL) (for 25 μL of inner phase)	Final concentration
Buffer (acetate and NaCl)	50 and 750 mM	5	10 and 150 mM
Sucrose	1000 mM	5	200 mM
Urease	20 mg mL^{-1}	4.4	3.5 mg mL^{-1}
Water	-	10.6	-

DNA-nanoswitch receivers:

	Stock concentration	Added volume (μL) (for 25 μL of inner phase)	Final concentration
Buffer (acetate and NaCl)	50 and 750 mM	5	10 and 150 mM
Sucrose	1000 mM	5	200 mM
DNA	100 μM	2.5	10 μM
Water	-	12.5	-

Acetylcholinesterase-based senders:

	Stock concentration	Added volume (μL) (for 25 μL of inner phase)	Final Concentration
Buffer (acetate and NaCl)	50 and 750 mM	5	10 and 150 mM
Sucrose	1000 mM	5	200 mM
Enzyme	10 mg mL^{-1}	4.4	1.8 mg mL^{-1}
Water	-	10.6	-

2. Add a schematic to Figure 2 to clearly convey the experimental design and processes described in this work.

As wisely suggested by the reviewer, we have added a schematic to Figure 2 to clearly convey the experimental design and processes. The corresponding paragraph and figure caption have also been adjusted to clarify the experimental design and processes.

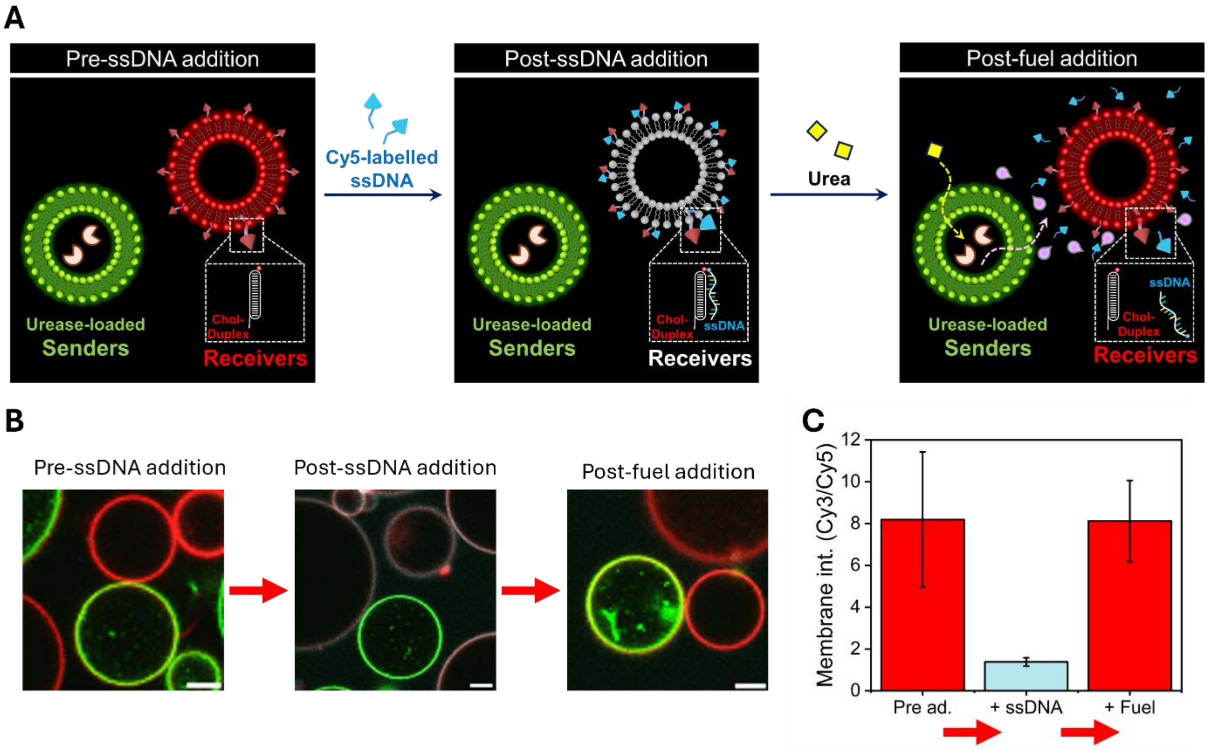

Figure 2. Capture and release of ssDNA from the membrane of duplex-functionalized receivers in an artificial cell consortium. **A)** Schematic representation of the experimental design and sequential processes, and **B)** resulting micrographs. Pre-ssDNA addition: urease-loaded senders (green) and receivers (red) functionalized with cholesterol (chol)-duplex are co-incubated. Post-ssDNA addition: after addition and incubation with ssDNA (20 min), the

formation of a triplex nanostructure on the membrane of duplex-functionalized receivers induces the decrease of the Cy3/Cy5 signal. Post-fuel addition: after subsequent incubation (70 min) upon addition of urea (25 mM), senders induce the release of ssDNA from receivers, leading to the recovery of the Cy3 fluorescence. Scale bars represent 5 µm. **C)** Output (Cy3/Cy5 membrane intensity ratio) quantification for the different conditions. Data plotted as mean ± s.d. (N ≥ 12 GUVs).

3. Since the microfluidic channel is crucial for spatiotemporal signal communications, please include a photograph of the channels in the supplementary information (SI) and provide detailed parameter information in the experimental section.

As suggested, we have included a photograph and details of the channel in the SI.

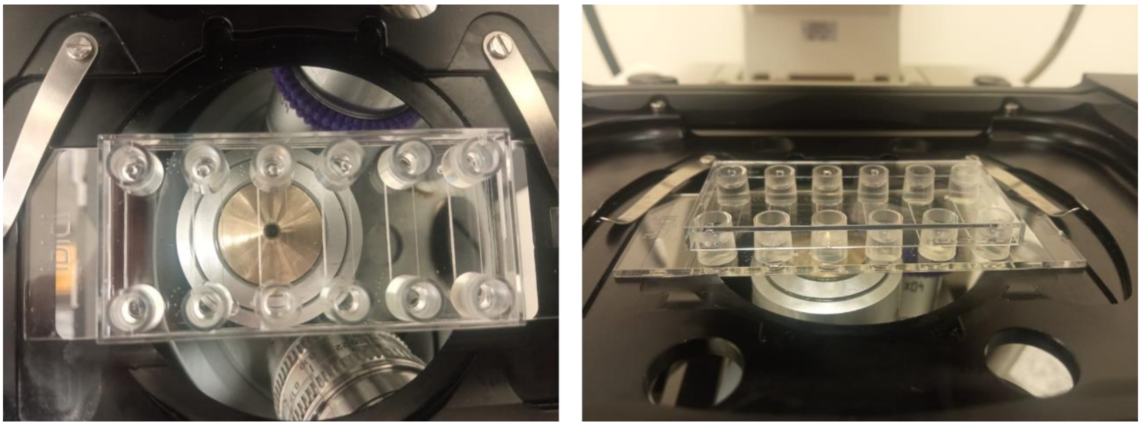

Figure S16. Photographs of the microfluidic channels employed in our study (mounted on the confocal microscope). The commercial device (from Ibidi) consists of a microscope slide (glass bottom) with six parallel channels with the following dimensions: length = 17 mm, width = 3.8 mm, height = 0.54 mm; channel volume = 40µL, volume per reservoir = 60 µL.

4. To emphasize "Spatiotemporal Communication," please supplement Figure 1 with time-dependent fluorescence imaging for a single vesicle at a fixed position, demonstrating the pH-dependent DNA conformation change in receiver vesicles.

As suggested, we have added time-dependent fluorescent imaging of a single receiver vesicle to Figure 1 (and additional ones in Figure S11). We confirmed that the pH of the external phase changed from 5 to 9 during incubation, correlating with the change in the DNA nanostructure.

In addition, following the reviewer's suggestion, also in our revised manuscript we provide videos of time-dependent DNA conformation change in artificial cell consortia (Videos S1, S2 and S3).

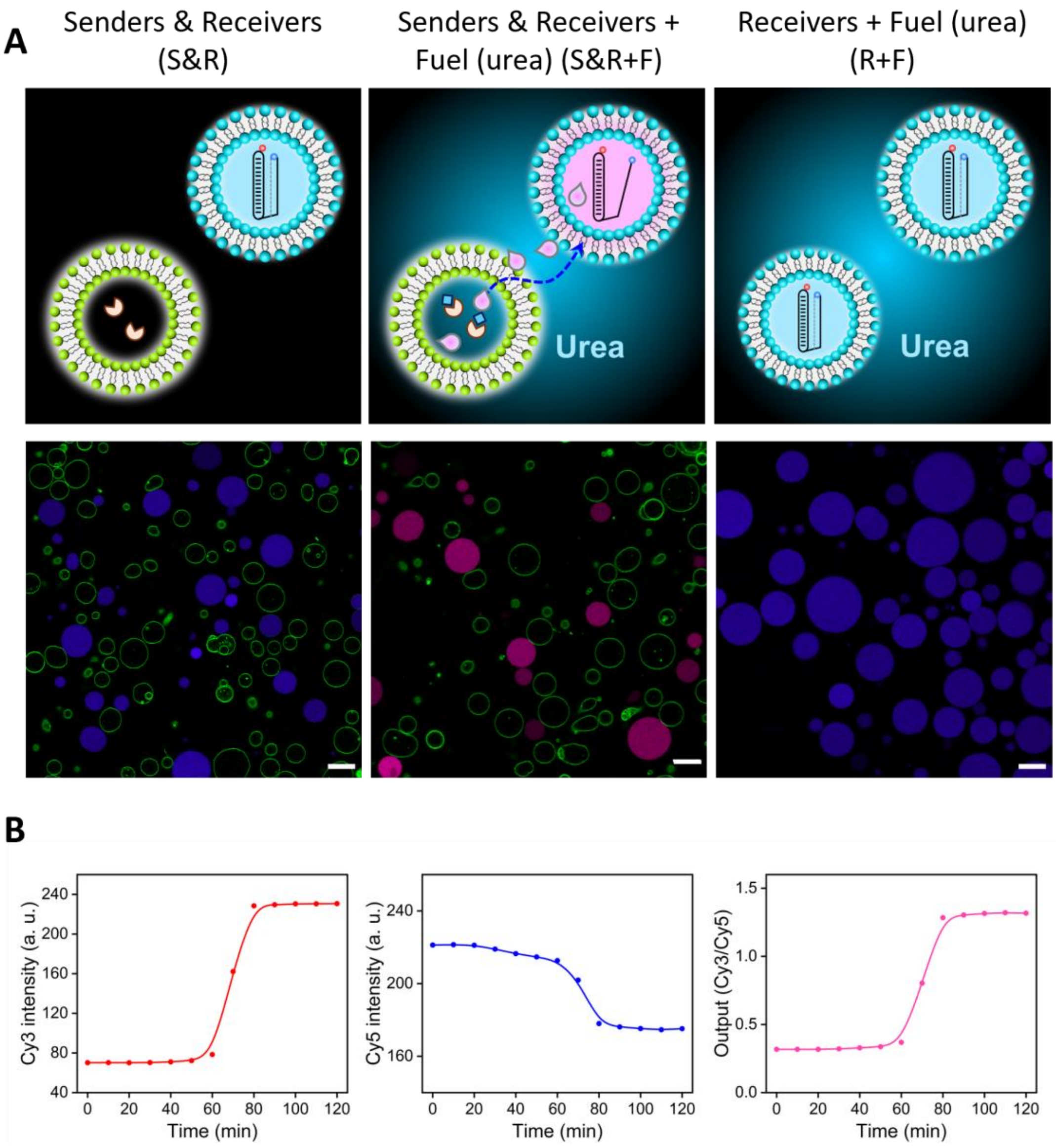

Figure 1. Chemical communication experiments between senders (urease-loaded, green membrane labelling) and receiver GUVs (DNA-loaded) in close proximity. A) Schematic and corresponding micrographs. Images show the merge of three channels: green (sender GUVs), red and blue (Cy3 and Cy5 in receiver GUVs, respectively) – which results in either blue (non-activated) or pink (activated) receivers. (left) In the absence of urea, the entrapped DNA nanostructure remains in the triplex state (blue); (middle) with input of urea (25 mM), ammonia-induced pH increase leads to the switching of the DNA nanostructure to the duplex conformation (pink); (right) in the absence of senders, there is no response. Scale bars represent

20 µm. **B)** Time-dependent fluorescence profiles of a selected (DNA-loaded) receiver vesicle upon signaling from sender vesicles in the presence of urea. Changes in Cy3 and Cy5 emission demonstrate conformational change of the DNA nanostructure. Profiles of additional vesicles can be found in Figure S11.

5. Cite relevant literatures associated with protocell signal communications or oscillating pH regulation, specifically: Chem. Sci., 2019, 10, 9446-9453; Small Methods, 2023, 7, e2300042; Chem 2024, 10, 600–614.

As suggested by the referee, we have added additional references as follows:

"Efforts to establish communication between artificial cells have recently been reviewed.[14]"

"In a recent report, the exchange of substrates between enzyme-functionalized liposomes was leveraged to induce enhanced motility of receivers.[24]"

"Control over protocell arrangements into microarrays has been achieved using acoustic trapping.[28] Recently, oscillatory behavior has been demonstrated on buoyant enzyme-powered protocells,[29] and on communities of artificial and bacterial cells able to drive environmental pH changes.[30]"

14. Wang, Z.; Zhang, M.; Zhou, Y.; Zhang, Y.; Wang, K.; Liu, J. Coacervate Microdroplets as Synthetic Protocells for Cell Mimicking and Signaling Communications. *Small Methods* **2023**, *7* (12), 2300042.

24. Tseng, Y. C.; Song, J.; Zhang, J.; Shandilya, E.; Sen, A. Chemomechanical Communication between Liposomes Based on Enzyme Cascades. *J Am Chem Soc* **2024**, *146*, 16097–16104.

28. Wang, X.; Tian, L.; Du, H.; Li, M.; Mu, W.; Drinkwater, B. W.; Han, X.; Mann, S. Chemical Communication in Spatially Organized Protocell Colonies and Protocell/Living Cell Micro-Arrays. *Chem. Sci.* **2019**, *10* (41), 9446–9453.

29. Peschke, P.; Kumar, B.; Walther, T.; Patil, A.; Mann S. Autonomous Oscillatory Movement of Sensory Protocells in Stratified Chemical Media. *Chem* **2024**, *10*, 600–614.

30. Li, S.; Zhao, Y.; Wu, S.; Zhang, X.; Yang, B.; Tian, L.; Han, X. Regulation of Species Metabolism in Synthetic Community Systems by Environmental PH Oscillations. *Nat. Commun.* **2023**, *14* (1), 1–10.

Reviewer 2:

This manuscript aims to advance the communication between artificial cells. They present an overall interesting idea but the manuscript is very condensed and full of fancy sounding terms, which make the reading of the manuscript difficult. The introduction would also benefit from

some more depth of such an active field. Overall, the manuscript could become publishable in this journal after major revisions. In particularly, the authors have provide stronger argumentation while their results that are largely based on the diffusion of small molecules are illustration control and communication.

We thank the reviewer for their positive assessment and suggestions for improvement. We have revised the manuscript considering their suggestions. Regarding the introduction, we have added additional references and corresponding discussions.

1. The text that goes with Figure 2 is too short and it is difficult to understand what the authors want to illustrate and why. Where is the SSDNA? Outside of the GUV?

We thank the reviewer for pointing this out. The explanation and argumentation of experiments regarding Figure 2 have been extended for clarity. Indeed, the ssDNA is added to the external medium. In addition, we have edited Figure 2 to illustrate the experimental design and processes.

2. Is it really fair to call the results in Figure 3 spatiotemporal communication since it is simple small molecule diffusion?

Considering the reviewer's comment, we have rephrased the introduction of these results as follows: "*Next, to demonstrate the ~~potential for spatiotemporal communication~~ possibility to activate receivers at different distances from senders (based on the diffusion of a secreted chemical signal), we employed a microfluidic channel device as depicted in Figure 3 and S16 (dimensions 17 x 3.8 x 0.54 mm).*"

3. In Figure 4, does N=14 mean that 14 GUVs were analyzed? That seems a very small set since the authors have no problem to create large populations. Are there independent repeats to illustrate the variability in encapsulation? Can the authors control their system by controlling how much material they encapsulate?

Indeed, N=14 refers to the number of analyzed GUVs (we have clarified this in the manuscript). Micrographs were analyzed using Fiji (ImageJ) using standard protocols. To quantify the fluorescence output, individual GUVs were randomly selected as region of interest and

manually tracked over subsequent frames while correcting for slight variations in position. Although automatic selection could be performed using Fiji algorithms, in our case we found the manual tracking more efficient to discriminate between different types of GUVs in close proximity. As mentioned by the reviewer, samples contained a large number of GUVs yet we kept the number of analyzed GUVs to N=14 for convenience as it gave a representative overview of the sample and increasing the number of analyzed GUVs did not have a significant effect on the mean and standard deviation of each time point. This information has been added to the manuscript.

In our study, experiments were independently repeated at least two times. In every repeat, similar trends were obtained. Following the reviewer's comment, to show the variability in different GUV batches, we additionally provide Figure S19:

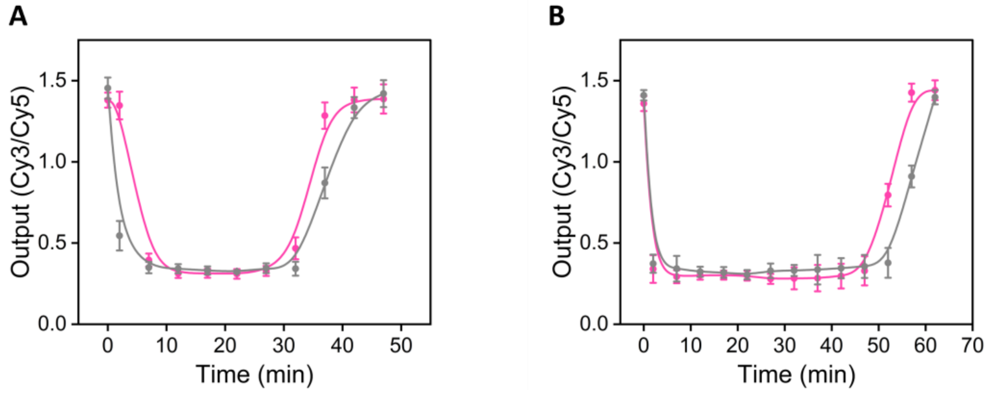

Figure S19. Comparative time-dependent output from two independent repeats (pink and grey lines, respectively) of dynamic reorganization of DNA nanostructures in consortia with two antagonistic sender populations. A) Equal proportion of sender population-1 (urease-loaded) and sender population-2 (acetylcholinesterase-loaded). B) Major proportion of population-2 (acetylcholinesterase-loaded) *vs*. population-1 (urease-loaded). Data plotted as mean ± s.d. (N = 14 GUVs). As observed, similar trends were obtained in independent repeats (pink and grey).

In addition, in this revised version, we demonstrate in Figure S4 that is possible to control the signaling rate by adjusting the concentration of enzyme added in the inner phase during the GUV preparation process.

4. ‘Such transient spatiotemporal activation patterns are reminiscent of cellular signaling in multicellular organisms, where molecules such as hormones and morphogens display dynamic

concentration gradients that drive different biological processes in receivers.' is in my option a very strong overstatement to what the authors show.

Following the reviewer's comment, we have rephrased such statement as follows:

"Transient spatiotemporal activation patterns are characteristic of cellular signaling in multicellular organisms, where molecules such as hormones and morphogens display dynamic concentration gradients that drive various biological processes in receivers.[9] In this regard, the creation of spatiotemporal patterns using artificial cells is an important step toward mimicking the complexity of natural cellular signaling."

5. The signaling front should be included in Fig 5a. Alternatively, all the sub-figs in 5 should have a illustration. The text is very condensed, which makes it difficult to understand what is actually happening. I am sure I understand how this experiments show transient spatiotemporal activation patterns.

As suggested by the reviewer, we have edited Figure 5a for a better understanding and to also include the signaling front. The corresponding text has also been revised for clarity.

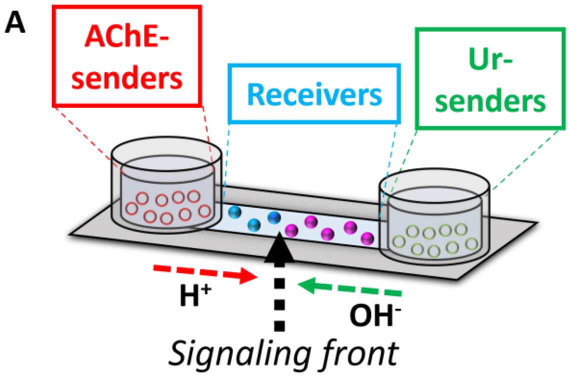

Figure 5. Spatiotemporal communication drives transient activation of receivers. A) Schematic representation of the set-up, where receivers are placed along a microfluidic channel. Acetylcholinesterase (AChE)-loaded senders and urease (Ur)-loaded senders are located at opposite ends. The system is initially set at pH 9, at which the DNA nanostructure is in the duplex state (pink). Signaling from the acid-producing AchE-senders induces the transition of receivers to the triplex state (blue). The position of the signaling front (furthest receiver in the triplex state (blue)) evolves with time, advancing in the channel during the first hours and receding as base-signaling from Ur-senders increases.

6. The manuscript would also benefit from a more elaborate statement on how this would be eventually useful for creating avenues for engineering.

As suggested by the reviewer, we have added the following statement:

"*The design of spatiotemporal patterns based on chemical communication between artificial cells holds potential in different areas, such as the development of advanced drug delivery systems and smart materials. For instance, senders could potentially sense the levels of certain biomarkers in one area and emit diffusive chemical signals to dynamically activate drug delivery by distal receivers located in target tissues. Different actuating mechanisms could be implemented in receivers; in particular, changes in DNA conformation could activate protein synthesis or induce membrane deformations. In addition, dynamic communication mechanisms could be integrated into artificial tissues to develop dynamic smart materials that exhibit spatiotemporal changes in response to environmental conditions*."
